# Supplementary material for: Autoimmune Renal Disease Is Exacerbated by S1P-Receptor-1-Dependent Intestinal Th17 Cell Migration to the Kidney
Source: Immunity. 2016 Nov 15;45(5):1078–92. doi: 10.1016/j.immuni.2016.10.020 (PMC6381450; doi:10.1016/j.immuni.2016.10.020)
Supplement: Document S1. Supplemental Experimental Procedures and Figures S1–S7 [file mmc1.pdf]

Immunity, Volume *45*

Supplemental Information

Autoimmune Renal Disease Is Exacerbated by S1P-Receptor-1-Dependent Intestinal Th17 Cell Migration to the Kidney

Christian F. Krebs, Hans-Joachim Paust, Sonja Krohn, Tobias Koyro, Silke R. Brix, Jan-Hendrik Riedel, Patricia Bartsch, Thorsten Wiech, Catherine Meyer-Schwesinger, Jiabin Huang, Nicole Fischer, Philipp Busch, Hans-Willi Mittrücker, Ulrich Steinhoff, Brigitta Stockinger, Laura Garcia Perez, Ulrich O. Wenzel, Matthias Janneck, Oliver M. Steinmetz, Nicola Gagliani, Rolf A.K. Stahl, Samuel Huber, Jan-Eric Turner, and Ulf Panzer

Patients	Age (years)	Sex	ANCA type	Creatinine (mg/dL)	Proteinuria	Immunosuppression prior to biopsy
1	73	f	pANCA (MPO)	0.7	0.8 g/d	Steroids
2	41	m	cANCA (PR3)	1.2	1.0 g/d	MTX / Steroids
3	51	m	pANCA (MPO)	1.6	0.8 ratio	None
4	63	m	pANCA (MPO)	1.8	1 ratio	AZA / Steroids
5	74	m	pANCA (MPO)	2.6	0.5 ratio	None
6	66	m	pANCA (MPO)	9.1	1.5 ratio	Steroids
7	55	f	pANCA (MPO)	1.6	NA	RTX / Steroids
8	64	m	cANCA (PR3)	3.2	0.25 g/d	AZA / Steroids

Figure S1. Related to Figure 1. Baseline characteristics of patients with ANCA-GN at the time of biopsy. ANCA-GN = anti-neutrophil cytoplasmic antibody-associated glomerulonephritis; pANCA = perinuclear ANCA; MPO = myeloperoxidase; cANCA = cytoplasmic ANCA; PR3 = proteinase 3; m = male; f = female.; MTX = methotrexate; AZA = azathioprin; RTX = Rituximab; NA = not available.

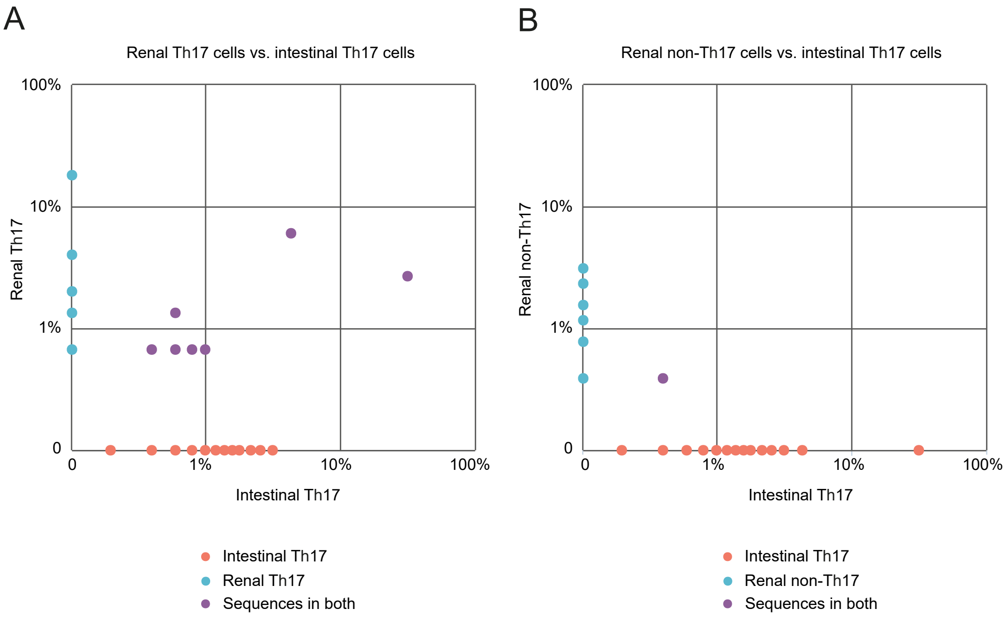

Figure S2. Related to Figure 2. Renal Th17 cells share more *TCRβ* sequences with intestinal Th17 cells than renal non-Th17 cells. cGN was induced in IL-17A-fate reporter mice (*Il17a*^{Cre} x *R26R*^{eYFP}). At day 10 renal Th17 cells (YFP^{+}), renal non-Th17 cells (YFP^{-}) and small intestinal Th17 cells (YFP^{+}) were FACS sorted, DNA was extracted and samples were analyzed for *TCRβ* sequences using the ImmunoSEQ platform (n=3). (A) Representative dot-plot diagram showing the percentage of individual clones in intestinal Th17 cells (orange), renal Th17 cells (blue) and those present in both populations (violet). (B) Dot plot showing the percentage of individual clones in intestinal Th17 cells (orange), renal non-Th17 cells (blue) and those present in both populations (violet).

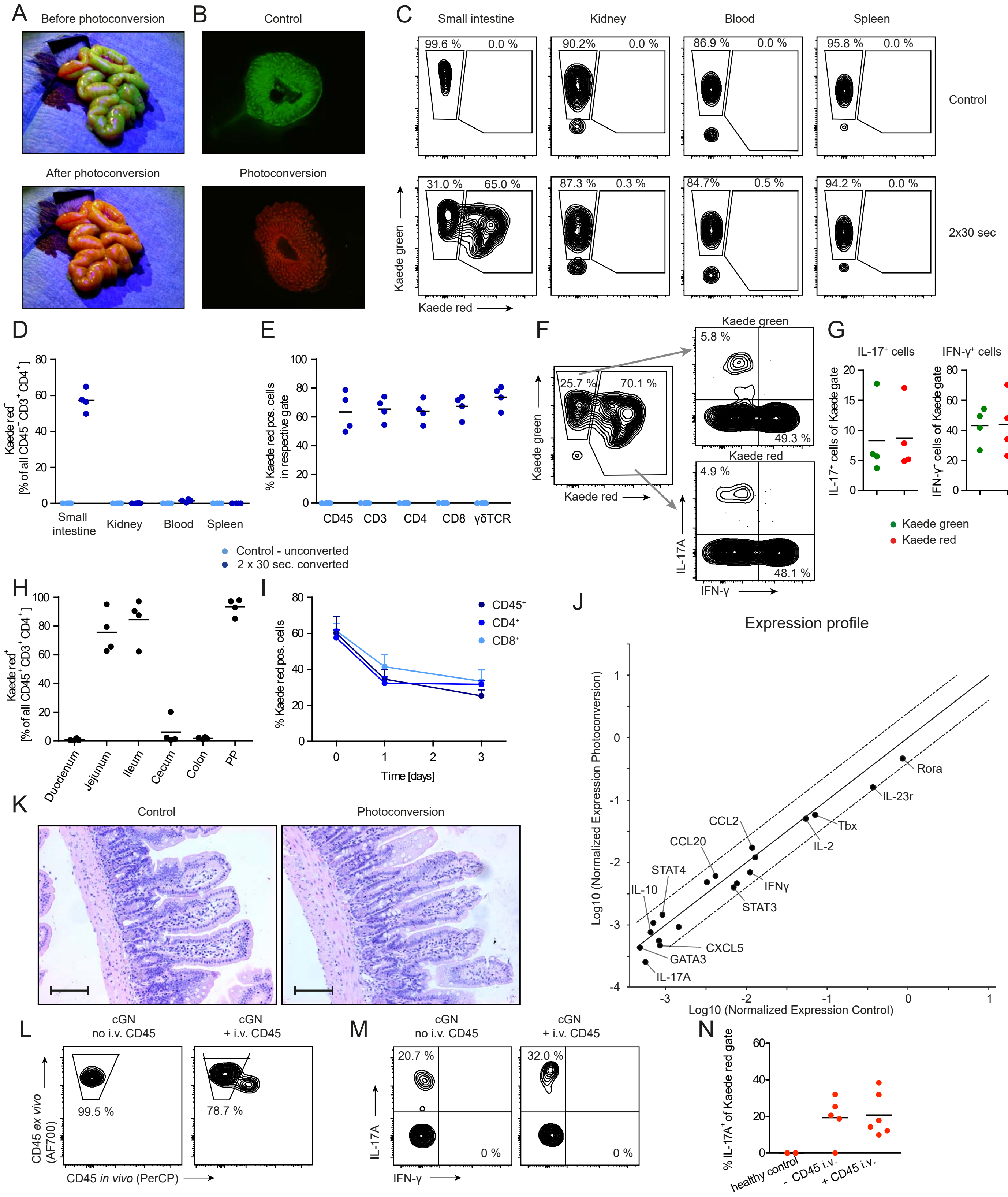

Figure S3. Related to Figure 3. Labeling of intestinal cells in photoconvertible *Kaede*-transgenic mice. (A) Photographs before and after photoconversion of cells in the small intestine using *Kaede* mice. (B) Confocal microscopy of the small intestine before and after photoconversion in *Kaede* mice. (C) Flow cytometry for Kaede green (unconverted) and Kaede red (converted) cells of small intestine lamina propria, kidney, blood and spleen from *Kaede*-tg mice directly after photoconversion of cells in the small intestine and *Kaede*-tg mice without photoconversion (control). (D) Quantification of photoconversion rate in $CD3^+CD4^+$ T cells (Kaede red^+ cells) in *Kaede*-tg mice directly after photoconversion of cells in the small intestine (dark blue) and *Kaede*-tg control (light blue). (E) Rate of photoconversion (Kaede red^+) in CD45, CD3, CD4, CD8 and γδ T cells from small intestine after photoconversion of the small intestine (dark blue) and control (light blue). (F) Kaede $green^+$ and Kaede red^+ were analyzed for IL-17A and IFN-γ expression. (G) Quantification of IL-17A and IFN-γ in Kaede $green^+$ and Kaede red^+ cells. (H) Quantification of the distribution of Kaede red^+ cells in the intestine directly after photoconversion of the small intestine in *Kaede*-tg mice. (I) Pulse-chase analysis of Kaede red^+ cells in the small intestine after photoconversion of the small intestine in *Kaede*-tg mice (n=3-4 per group). (J) Cytokine and chemokine expression profile from *Kaede*-tg mice 1 day after photoconverion of the small intestine and untouched control mice. (K) HE staining of jejunum of *Kaede*-tg mice 1 day after photoconversion of the small intestine and of untouched control mice. To discriminate between renal and intravascular T cells, mice recieved 5 µg PerCP labelled CD45 antibodies intraveneously 3 min prior to analysis. (L) Analysis of $CD4^+$ T cells from the kidney. (M+N) Analysis of renal $CD4^+$ T cells for IL-17 and IFN-γ expression in mice +/- intraveneously CD45-PerCP. Data are representative of three independent experiments. Scale bar 200 µm.

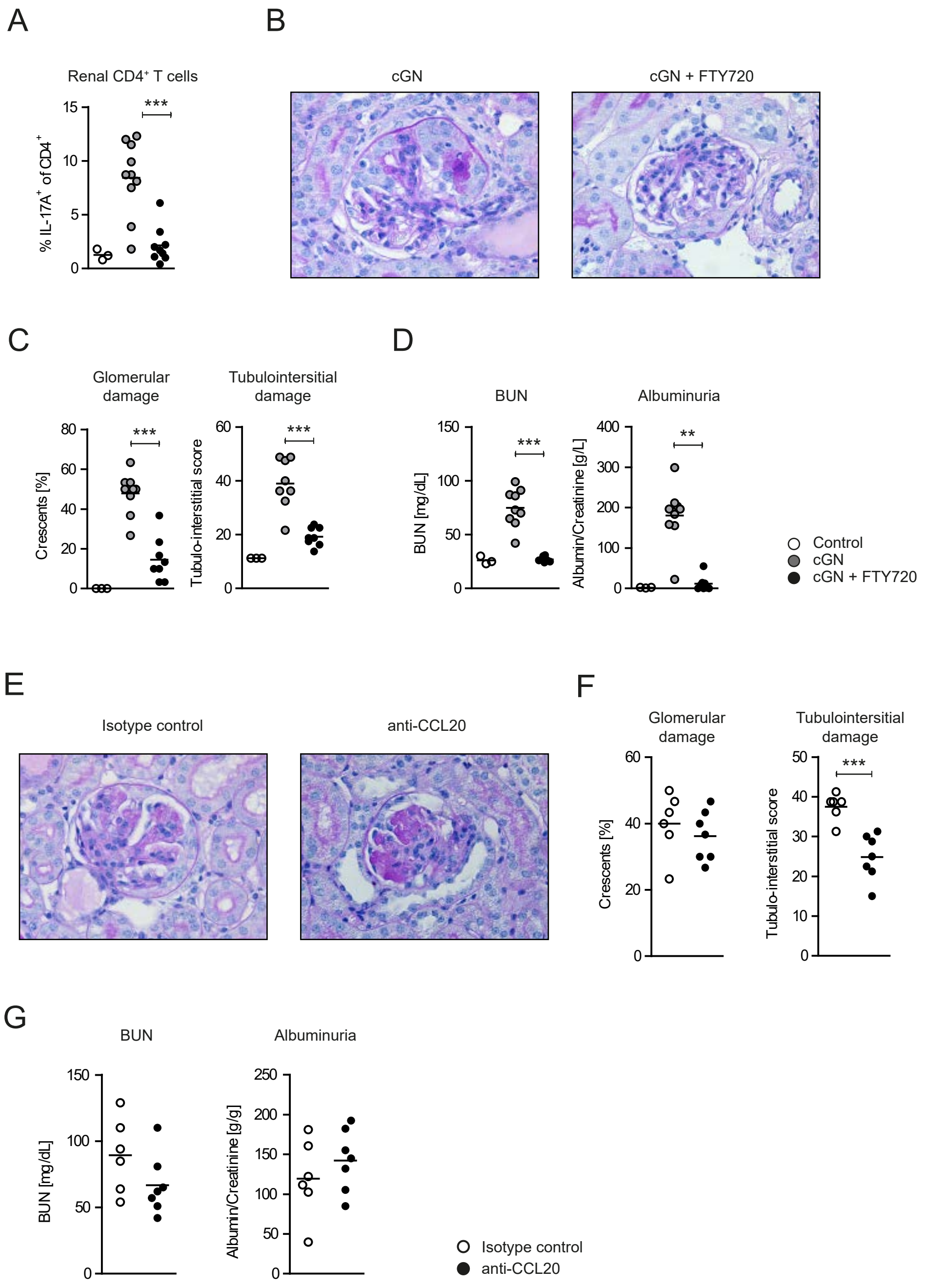

Figure S4. Related to Figure 4. FTY720 reduces renal Th17 cell infiltration and subsequent tissue injury in cGN while CCL20 neutralization did not affect the clinical cours of cGN. (A) After induction of cGN mice were treated with FTY720 via drinking water and analyzed for Th17 cells in the kidney at day 8. (B) PAS-stained tissue sections of renal cortex. (C) Quantification of renal tissue damage (glomerular crescents and tubulointerstitial score) and (D) kidney function (blood urea nitrogen, BUN and albuminuria) were quantified. (E-G) cGN was induced in mice. From day 4 until day 7 mice received anti-CCL20 mab or isotype control. Photoconversion was performed at day 4. (E) PAS-stained tissue sections of renal cortex. (F) Renal tissue damage was quantified (glomerular crescents and tubulointerstitial score) and (G) renal function was measured (blood urea nitrogen, BUN and albuminuria). Data are representative of two (A-D) or three (E-G) independent experiments. ** $P<0.01$, *** $P<0.001$.

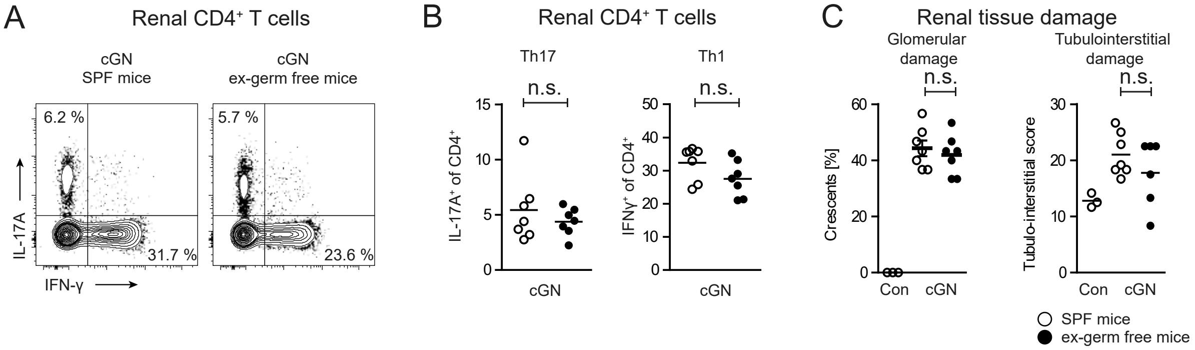

Figure S5. Related to Figure 5. Renal Th17 response was not different between nephritic mice and conventionally colonized ex-germ free mice. (A) IL-17A and IFN-γ expression by flow cytometry of renal CD4$^+$ T cells after induction of cGN in SPF mice and ex-germ free mice reconstituted with SPF microbiota. (B) Quantification of IL-17A and IFN-γ expression. (C) Quantification of glomerular crescent formation and tubulointerstitial damage. Data are representative of two independent experiments. Symbols represent individual data points with the mean as a horizontal line.

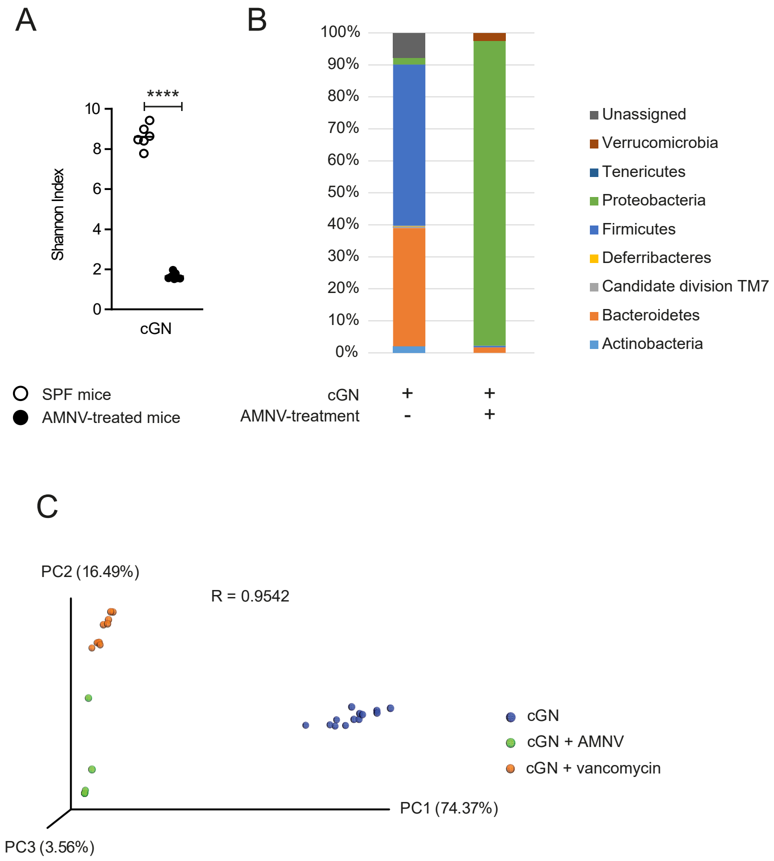

Figure S6. Related to Figure 5. Effect of antibiotic treatment on intestinal microbiota. (A) Analysis of alpha diversity (shannon index) of microbiota from large intestine of mice with AMNV-treatment (ampicillin, metronidazole, neomycin, vancomycin) and SPF control mice. (B) Abundance of bacteria on phylum level in mice after AMNV-treatment and control. (C) Beta diversity analysis of the datasets. 3D Principle component analysis plots showing the relation of the datasets using weighted UniFrac metric. Individual datasets are represented as colored spheres. Analysis of similarity (ANOSIM) tests were applied showing that the microbiota profiles from the datasets are highly significant (cGN: n=13; cGN + AMNV: n=11; cGN + vancomycin: n=10). Data are representative of two independent experiments. **** P<0.0001. The R-statistics was calculated as 0.9542.

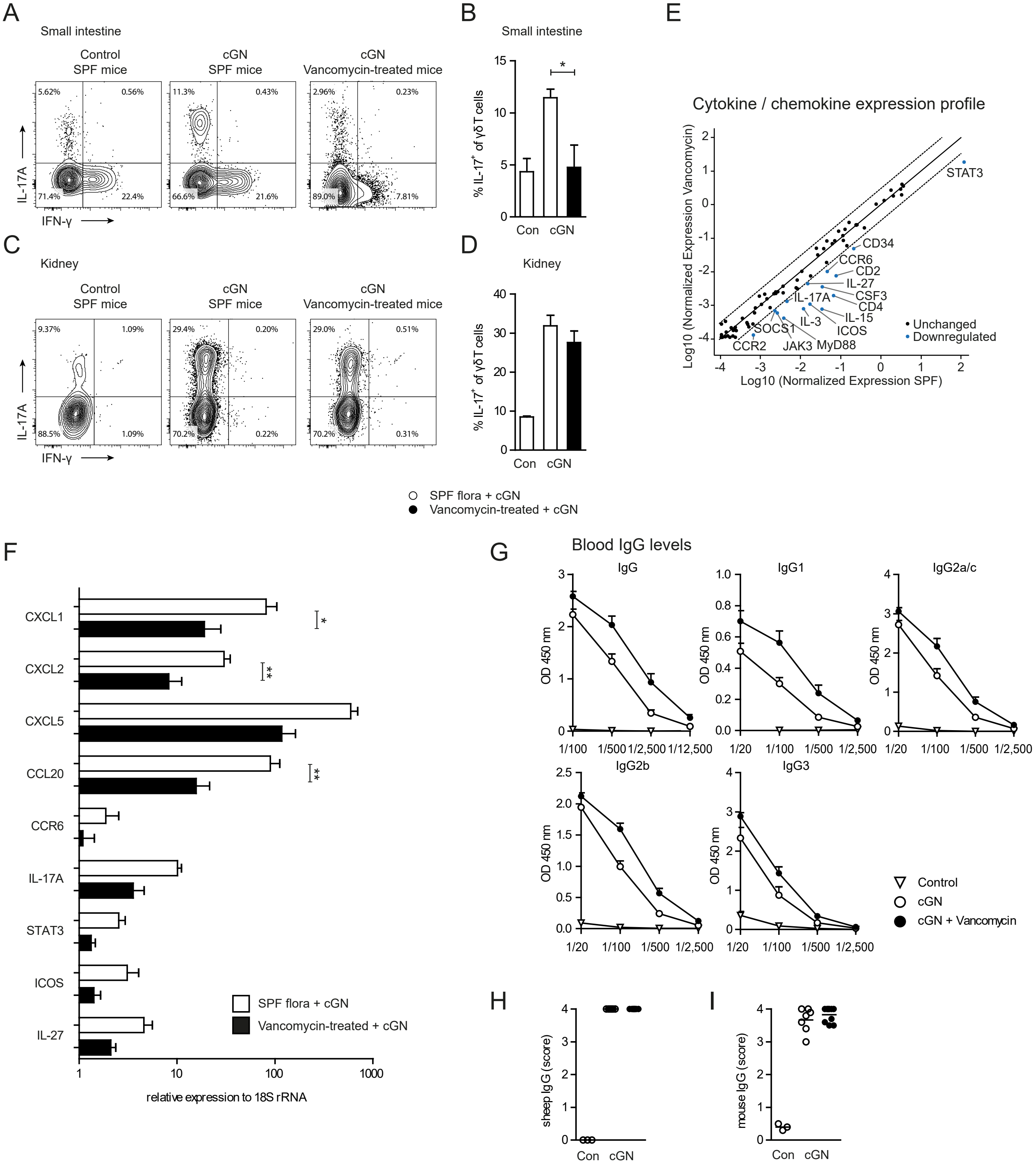

Figure S7. Related to Figure 7. Vancomycin-treatment reduces IL-17A expression by intestinal but not renal γδ T cells and affects renal cytokine and chemokine expression in cGN indendent of the humoral immune response. γδ T cells from the small intestine (A-B) and the kidney (C-D) of mice with cGN were analyzed for IL-17 and IFN-γ expression by flow cytometry. (E+F) Mice were treated for 4 weeks with vancomycin via the drinking water prior to induction of cGN. (E) Cytokine and chemokine expression profile of renal cortex from SPF mice with cGN and vancomycin-treated mice with cGN. (F) RT-PCR analysis of renal cortex as indicated (n=5-6 per group). (G) ELISA of circulating serum mouse anti-sheep total IgG-, IgG1-, IgG2a/2c-, IgG2b-, and IgG3-levels at different dilutions from controls (n=3), nephritic wildtype (n=7), and vancomycin treated nephritic mice (n=10) ten days after induction of cGN. (H) Quantification of glomerular sheep IgG- and (I) glomerular mouse IgG-deposition from controls (n=3), nephritic wildtype (n=7), and vancomycin treated nephritic mice (n=10). Data are representative of three independent experiments. * P<0.05, ** P<0.01.

SUPPLEMENTAL EXPERIMENTAL PROCEDURES

Generation of nephrotoxic serum

To generate nephrotoxic serum, murine renal cortex was homogenized by magnetic beads using a Tissue Lyser II (Qiagen, Hilden, Germany) and sonicated subsequently using a Bandelin Sonopuls (Bandelin, Berlin, Germany) at 100%. Pellets were resuspended in PBS and used for immunization of sheep. Immunization of sheep was performed 4-times within 3 months at Eurogentec (Cologne, Germany). Sheep immunoglobulin fraction was enriched in two precipitation steps with ammonium sulfate at a final concentration of 50% and dialyzed against phosphate buffered saline (PBS).

Morphological analyses

Paraffin-embedded sections (2 µm) were stained with an antibody directed against GR-1 (Ly6 G/C; NIMP-R14, Hycult Biotech, Uden, Netherland). Tubulointerstitial GR-1^{+} cells in 20 low-power fields (magnification ×200) were counted. For the evaluation of Kaede photoconversion in cells of the small intestine, tissue sections were subjected to confocal imaging without further preparation. Kidney samples were snap-frozen in Tissue-Tec and stored at -80 °C. Cryosections were cut at 12 mm on a Leica model CM1850 freezing microtome, stained with anti-CD3 and analyzed by confocal imaging. All slides were evaluated under an Axioskop light microscope and photographed with an Axiocam HRc (Zeiss) using the ZEN software or by confocal microscopy with an A1R using NIS-Element software (Nikon).

T cell receptor sequencing

At day 10 after induction of cGN in *Il17a* fate reporter mice (*Il17a*^{Cre} x *R26R*^{eYFP}) renal Th17 cells (YFP^{+}; 3-7.5 x 10^{4} cells), renal non-Th17 cells (YFP^{-}; 10-50 x 10^{4} cells) and small intestinal Th17 cells (YFP^{+}; 1-2 x 10^{4} cells) were FACS sorted. DNA extraction was performed using the QIAamp DNA Micro Kit (Qiagen) according to the manufacturer's instructions. TCRβ-chain sequencing was conducted by Adaptive Biotechnologies (Seattle, WA, USA) based on the ImmunoSEQ platform (http://www.immunoseq.com) (Becattini et al., 2015). Samples with > 80 productive TCR sequences were taken into the final analysis.

16S rRNA amplicon library preparation and MiSeq sequencing

DNA was automatically extracted from mouse feces using QiaSymphony (Qiagen). Briefly, mouse feces were resuspendend in 1ml extraction buffer; 200 µl were used in the extraction protocol and DNA was eluted in 100µl volume. Amplicons were generated using the following degenerate primers containing the Illumina adapter consensus sequence F (5'- TCGTCGGCAGCGTCAGATGTGTATAAGAGACAGCCTACGGG NGGCWGCAG-3') and R (5'-GTCTCGTGGGCTCGGAGATGTGTATAAGAGACAGGACTAC HVGGGTATCTAATCC-3`) as recently described (Klindworth et al., 2013). Detailed description of the protocol is provided by Illumina (http://www.illumina.com/content; 16s-metagenomic-library-prep-guide-15044223-b.pdf). Illumina Nextera XT Index Kit was used for multiplexing. Barcoded libraries were quantified using the Qubit dsDNA HS Assay Kit (Life Technologies) and subsequently pooled. The libraries were sequenced by 2 x 500 bp PE sequencing on the MiSeq platform.

Bioinformatic processing

FastQC (Babraham Bioinformatics, Babraham Institute, UK) was used to analyze the average quality scores of each sample before and after paired reads. The paired ends in each sample were joined and all sequences less than 250 bp and/or with a Phred score <33 were discarded. Further quality filtering was applied using QIIME (Caporaso et al., 2010) (at Phred >= Q20). QIIME version 1.7 was used to perform OTU clustering and alpha and beta diversity analysis. Chimera filter was applied using usearch8.1. All sequences were clustered based on 97% similarity to reference sequences. The reads railing to meet the 97% similarity were clustered de novo at 97% similarity. Taxonomy levels of representative sequences in the OTU table were assigned according to the SILVA database at 95% similarity. Alpha diversity based on Shannon diversity index was calculated. Beta diversity statistics (ANOSIM) was performed to determine whether differences between the distributions of the microbiota profiles from the three datasets were significant.

SUPPLEMENTAL REFERENCES

Becattini, S., Latorre, D., Mele, F., Foglierini, M., De Gregorio, C., Cassotta, A., Fernandez, B., Kelderman, S., Schumacher, T.N., Corti, D., *et al.* (2015). T cell immunity. Functional heterogeneity of human memory CD4(+) T cell clones primed by pathogens or vaccines. Science *347*, 400-406.

Caporaso, J.G., Kuczynski, J., Stombaugh, J., Bittinger, K., Bushman, F.D., Costello, E.K., Fierer, N., Pena, A.G., Goodrich, J.K., Gordon, J.I., *et al.* (2010). QIIME allows analysis of high-throughput community sequencing data. Nat Methods *7*, 335-336.

Klindworth, A., Pruesse, E., Schweer, T., Peplies, J., Quast, C., Horn, M., and Glockner, F.O. (2013). Evaluation of general 16S ribosomal RNA gene PCR primers for classical and next-generation sequencing-based diversity studies. Nucleic Acids Res *41*, e1.
