# Supplementary material for: Autoimmune Renal Disease Is Exacerbated by S1P-Receptor-1-Dependent Intestinal Th17 Cell Migration to the Kidney
Source: Immunity. 2016 Nov 15;45(5):1078–92. doi: 10.1016/j.immuni.2016.10.020 (PMC6381450; doi:10.1016/j.immuni.2016.10.020)
Supplement: Document S2. Article plus Supplemental Information [file mmc2.pdf]

Immunity

Article

Autoimmune Renal Disease Is Exacerbated by S1P-Receptor-1-Dependent Intestinal Th17 Cell Migration to the Kidney

Graphical Abstract

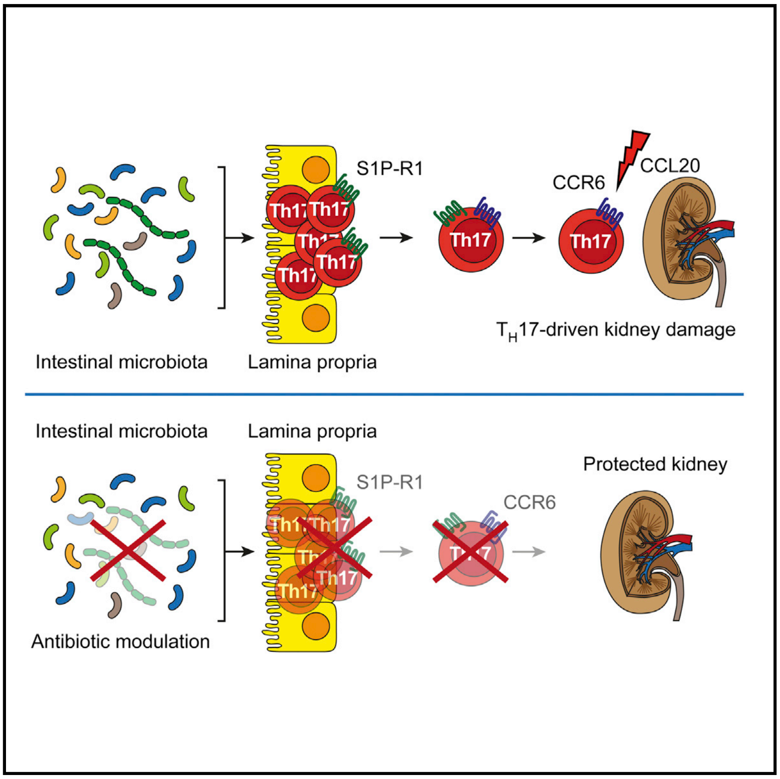

Highlights

- Pathogenic T_H17 cells migrate from the gut to the kidney in autoimmunity
- T_H17 cells egress the intestine in a S1PR1-dependent manner in glomerulonephritis
- Targeting microbiota-induced T_H17 cells ameliorates extraintestinal T_H17 responses

Authors

Christian F. Krebs, Hans-Joachim Paust, Sonja Krohn, ..., Samuel Huber, Jan-Eric Turner, Ulf Panzer

Correspondence

panzer@uke.de

In Brief

By photolabelling intestinal cells, Krebs and colleagues provide direct evidence that microbiota-induced T_H17 cells egress from the gut S1PR1-dependently and infiltrate the kidney via CCL20/CCR6 in immune-mediated diseases. This finding will build the basis for therapies targeting the intestinal T_H17 cell "reservoir" to treat extraintestinal T_H17 autoimmunity.

Krebs et al., 2016, Immunity *45*, 1078–1092
November 15, 2016 © 2016 The Authors. Published by Elsevier Inc.
http://dx.doi.org/10.1016/j.immuni.2016.10.020

Immunity
Article

Autoimmune Renal Disease Is Exacerbated by S1P-Receptor-1-Dependent Intestinal Th17 Cell Migration to the Kidney

Christian F. Krebs,[1] Hans-Joachim Paust,[1] Sonja Krohn,[1] Tobias Koyro,[1] Silke R. Brix,[1] Jan-Hendrik Riedel,[1] Patricia Bartsch,[1] Thorsten Wiech,[2] Catherine Meyer-Schwesinger,[1] Jiabin Huang,[3] Nicole Fischer,[3] Philipp Busch,[4] Hans-Willi Mittrücker,[5] Ulrich Steinhoff,[6] Brigitta Stockinger,[7] Laura Garcia Perez,[8] Ulrich O. Wenzel,[1] Matthias Janneck,[1] Oliver M. Steinmetz,[1] Nicola Gagliani,[4] Rolf A.K. Stahl,[1] Samuel Huber,[8] Jan-Eric Turner,[1] and Ulf Panzer[1,9,*]
[1]III. Medizinische Klinik
[2]Institut für Pathologie
[3]Institut für Medizinische Mikrobiologie, Virologie, und Hygiene
[4]Klinik für Allgemeinchirurgie
[5]Institut für Immunologie
Universitätsklinikum Hamburg-Eppendorf, 20251 Hamburg, Germany
[6]Philipps-Universität Marburg, Institut für Medizinische Mikrobiologie und Krankenhaushygiene, 35043 Marburg, Germany
[7]The Francis Crick Institute, Midland Road, London NW1 1AT, UK
[8]I. Medizinische Klinik, Universitätsklinikum Hamburg-Eppendorf, 20251 Hamburg, Germany
[9]Lead Contact
*Correspondence: panzer@uke.de
http://dx.doi.org/10.1016/j.immuni.2016.10.020

SUMMARY

Th17 cells are most abundant in the gut, where their presence depends on the intestinal microbiota. Here, we examined whether intestinal Th17 cells contribute to extra-intestinal Th17 responses in autoimmune kidney disease. We found high frequencies of Th17 cells in the kidneys of patients with antineutrophil cytoplasmatic antibody (ANCA)-associated glomerulonephritis. We utilized photoconversion of intestinal cells in *Kaede* mice to track intestinal T cell mobilization upon glomerulonephritis induction, and we found that Th17 cells egress from the gut in a S1P-receptor-1-dependent fashion and subsequently migrate to the kidney via the CCL20/CCR6 axis. Depletion of intestinal Th17 cells in germ-free and antibiotic-treated mice ameliorated renal disease, whereas expansion of these cells upon *Citrobacter rodentium* infection exacerbated pathology. Thus, in some autoimmune settings, intestinal Th17 cells migrate into target organs, where they contribute to pathology. Targeting the intestinal Th17 cell "reservoir" may present a therapeutic strategy for these autoimmune disorders.

INTRODUCTION

$CD4^+$ T cells are critical for defense against a wide array of invading microbes and pathogens but are also major drivers of autoimmune diseases. Based on their cytokine secretion profile and expression of specific transcription factors, $CD4^+$ T cells can be classified into functionally different subsets, e.g., Th1, Th2, Th17, and regulatory T cells (Tregs) (O'Shea and Paul, 2010). It was generally accepted that IFN-γ-expressing Th1 cells primarily initiate and perpetuate tissue damage in autoimmunity (Mosmann et al., 1986). This paradigm was challenged in 2005 by the discovery of a highly pathogenic IL-17-producing $CD4^+$ effector T cell subset, termed Th17 cells (Harrington et al., 2005; Park et al., 2005). Th17 cells are characterized by their key transcription factors RORγt and STAT3 (Ivanov et al., 2006; Nurieva et al., 2007), the production of the cytokines IL-17A, IL-17F, IL-22 and GM-CSF (Codarri et al., 2011; Zenewicz et al., 2007), and high expression of CCR6 (Acosta-Rodriguez et al., 2007). Today, their central role in the pathogenesis of several autoimmune diseases is clearly established (Gaffen et al., 2014).

Crescentic glomerulonephritis (cGN) is the most aggressive form of autoimmune kidney diseases that destroys kidneys over a period of days to weeks, leading to end-stage renal failure with associated high morbidity, mortality, and public health costs (Couser, 2012; Kurts et al., 2013). The infiltration of leukocytes, including T cells, and the proliferation of resident glomerular cells lead to the formation of glomerular crescents and a disrupted anatomical structure of the glomerulus, ultimately leading to loss of kidney function. Current treatment protocols are unspecific and hampered by toxic side effects that deteriorate patient outcome.

Recent studies have highlighted the substantial impact of the Th17 immune response in cGN (Kitching and Holdsworth, 2011; Kurts et al., 2013). This includes the identification and characterization of $CCR6^+$ IL-17-producing T cells in murine kidneys in experimental models of cGN (Paust et al., 2012; Turner et al., 2010), as well as evidence for the contribution of IL-17A, IL-17F, IL-17RA, IL-23p19, and RORγt to renal tissue injury in cGN (Paust et al., 2009; Ramani et al., 2014; Riedel et al., 2016; Steinmetz et al., 2011; Summers et al., 2009). Th17-cell-derived IL-17A and IL-17F promote the expression of chemokines such as CXCL1 and CXCL5 in the kidney and thereby drive

 © 2016 The Authors. Published by Elsevier Inc.
This is an open access article under the CC BY license (http://creativecommons.org/licenses/by/4.0/).

recruitment of neutrophils and other leukocyte subtypes, which mediate renal tissue destruction in cGN (Disteldorf et al., 2015; Turner et al., 2010). Although we are beginning to understand the effector functions of Th17 cells in the target tissue, the developmental origin of Th17 cells that infiltrate inflamed tissues, e.g., the kidney in glomerulonephritis, is still a matter of debate.

Under homeostatic conditions, Th17 cells are most abundant in the small intestinal lamina propria, and their presence in the gut of mice requires the colonization with specific adhesive microorganisms (Ivanov et al., 2009). Colonization of mice with segmented filamentous bacteria (SFB) results in the generation of SFB-specific Th17 cells (Yang et al., 2014). In addition to SFB, infection of mice with enterohemorrhagic *Escherichia coli* (EHEC) or *Citrobacter rodentium* results in the expansion of intestinal Th17 cells (Atarashi et al., 2015; Ivanov et al., 2009; Sano et al., 2015). In line with this, germ-free mice lack intestinal Th17 cells, and antibiotic treatment of mice can reduce intestinal Th17 cell frequencies (Atarashi et al., 2008; Ivanov et al., 2008; Rakoff-Nahoum et al., 2004). In addition, Th17 cells from lymphoid tissues preferentially home to the gut after transfer and are phenotypically almost indistinguishable from intestinal Th17 cells (Hirota et al., 2013). Th17 cells highly express CCR6, which orchestrates their trafficking to the small intestine (Esplugues et al., 2011) but also to sites of peripheral inflammation, such as the kidney in glomerulonephritis (Turner et al., 2010). Furthermore, organ-specific Th17 immune responses in experimental autoimmune encephalomyelitis (EAE) and arthritis are diminished in mice with reduced intestinal Th17 cells, i.e., in germ-free mice (Lee et al., 2011; Wu et al., 2010). Taken together, these observations indicate a close relationship of Th17 cells with the intestinal microbiota. However, the mechanisms by which microbiota-induced Th17 cells promote extra-intestinal Th17 immune responses remain to be fully elucidated.

Here, using transgenic mice that ubiquitously express the photoconvertible Kaede-protein, we directly demonstrated the migration of intestinal Th17 cells to the kidney in experimental cGN. Experiments in microbiota-manipulated mice underscore the concept of pathogenic Th17 cells migrating from the gut to the inflamed kidney. Our findings provide evidence supporting a role for intestinal Th17 cells in the exacerbation of GN and suggest that migration of intestinal Th17 cells may contribute to pathology in other autoimmune diseases.

RESULTS

Identification and Characterization of Th17 Cells in the Kidneys of Patients with ANCA-Associated cGN

Antineutrophil cytoplasmatic antibody (ANCA)-associated GN is the most common cause of cGN and is characterized by the formation of glomerular crescents (Figure 1A). This is associated with the infiltration of T cells and neutrophils (Figure 1B). To determine the composition of T cell subsets in ANCA-associated GN, we analyzed cells isolated from human renal biopsies by flow cytometry. The clinical characteristics of ANCA-GN patients included in this study are summarized in Figure S1. Th17 cells can be distinguished from other $CD4^+$ T cells via the expression of the transcription factor RORγt. We identified RORγt^+ cells in the kidneys of patients with ANCA-associated GN (Figures 1C and 1D). The frequency of renal $CD4^+ROR\gamma t^+$ T cells was high (about 30%) and increased compared to $CD4^+$ T cells from the peripheral blood (<3%) of patients with ANCA-GN (Figures 1C–1E). In control kidney samples derived from tumor nephrectomies, $CD4^+ROR\gamma t^+$ cells were detected at low frequencies (<3%, Figures 1E and 1F). RORγt expression by leukocytes isolated from inflamed kidneys was primarily allocated to $CD3^+$ T cells (~90%), and the majority of these were $CD4^+$ T helper cells (Figures 1C and 1G). We recently reported increased expression of CCR6 and its ligand CCL20 in the kidney of patients with ANCA-GN (Paust et al., 2015). In line with this finding, the majority of the $CD4^+ROR\gamma t^+$ Th17 cells in the kidney and blood expressed CCR6, supporting a role for this receptor in Th17 cell trafficking (Figure 1H).

Renal Th17 Cells Have Gut-Homing Properties in Experimental cGN

To further investigate the function and trafficking properties of Th17 cells, we used the well-characterized mouse model of cGN (Bollée et al., 2011; Krebs et al., 2013; Pisitkun et al., 2012; Tsuboi et al., 2008). The cGN model was induced by intraperitoneal (i.p.) injection of nephrotoxic sheep serum directed against the glomerular basement membrane (GBM). This prompted an adaptive immune response against the planted antigen, which resulted in the Th17-cell-dependent formation of glomerular crescents, tubulointerstitial injury, and loss of renal function (Figure 2A), resembling aspects of cGN in humans (Krebs et al., 2013; Paust et al., 2009; Steinmetz et al., 2011). In this model, *Il17a* fate reporter mice (*Il17a*^{Cre} x *R26R*^{eYFP}) were used to track Th17 cells (Hirota et al., 2011). $CD4^+$ Th17 cells in the kidney increased at day 3–5, peaked around day 7–10 after GN induction, and then declined (Figures 2B and 2C). Importantly, fate-mapped $eYFP^+$ Th17 cells almost uniformly expressed the signature cytokine IL-17A (Figure 2D), demonstrating the feasibility of this reporter system.

To evaluate the trafficking and homing properties of renal Th17 cells compared with other effector T cells, we sorted $eYFP^+$ Th17 cells and $eYFP^-$ $CD4^+$ T cells with an activated phenotype ($CD44^{high}$) from the kidney of nephritic *Il17a* fate reporter mice and co-transferred them at a 1:1 ratio into TCRα-deficient hosts lacking $\alpha\beta^+$ T cells (Figure 2E). $eYFP^+$ Th17 cells preferentially reconstituted (or were expanded in) the gut-associated tissues, such as the small intestinal lamina propria (SILP) and mesenteric lymph nodes, whereas $eYFP^-$ $CD44^{high}$ non-Th17 cells were preferentially found in peripheral lymph nodes (Figures 2F and 2G).

We next tested, vice versa, whether gut-derived Th17 cells are able to migrate to the kidney and, therefore, sorted small intestinal Th17 cells from *Il17a* fate reporter mice, transferred them into $Tcr\alpha^{-/-}$ animals and subsequently induced cGN. As shown in Figure 2H, gut-derived Th17 cells migrated to the kidney upon transfer. Moreover, they were sufficient to aggravate GN in T-cell-deficient animals (Figures 2I–2J).

To further examine the potential relationship of renal and intestinal Th17 cells, we analyzed the sequence of the CDR3 region of the *Tcrβ* gene of FACS-sorted $eYFP^+CD44^{high}$ cells (Th17) and $eYFP^-CD44^{high}$ cells (non-Th17) from the kidneys of nephritic *Il17a* fate reporter mice and compared those to intestinal Th17 cells sorted from the small intestine of the same mice, using the ImmunoSEQ platform. As shown in Figures 2K and S2, renal Th17 cells shared more *Tcrβ* sequences with intestinal Th17 cells

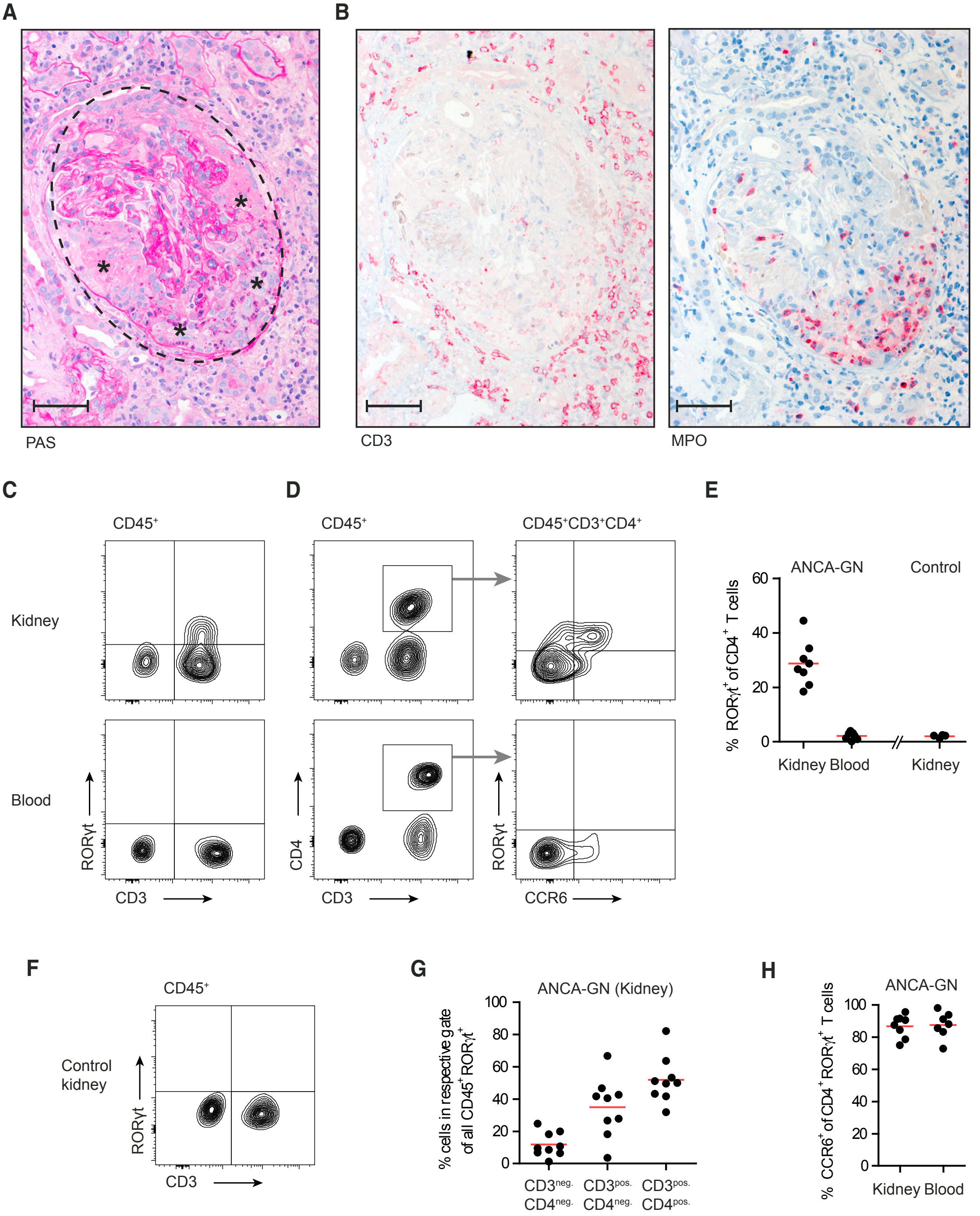

Figure 1. Identification and Characterization of Th17 Cells in the Kidneys of Patients with ANCA-Associated cGN

(A) PAS staining of a renal biopsy from a patient with ANCA-GN (glomerulus: dashed line; crescent: asterisked).
(B) Consecutive tissue sections were stained for CD3 (T cells) and MPO (neutrophils).
(C) Leukocytes from renal biopsies and peripheral blood samples from ANCA-GN patients were analyzed by flow cytometry.
(D) $CD3^{+}CD4^{+}$ cells were analyzed for expression of RORγt and CCR6.
(E) Quantification of $ROR\gamma t^{+}CCR6^{+}$ cells of all $CD4^{+}$ T cells in kidney and blood (ANCA-GN) and control biopsies (unaffected renal tissue of explanted kidneys after tumor nephrectomy).
(F) Flow cytometry of control biopsies ($CD45^{+}$ cells).
(G) Quantification of $CD45^{+}ROR\gamma t^{+}$ cells from the kidneys of patients with ANCA-GN as indicated.
(H) CCR6 expression of $ROR\gamma t^{+}CD4^{+}$ T cells from the kidney and peripheral blood (ANCA-GN). Symbols represent individual data points with the mean as a horizontal line. Scale bar, 30 μm. See also Figure S1.

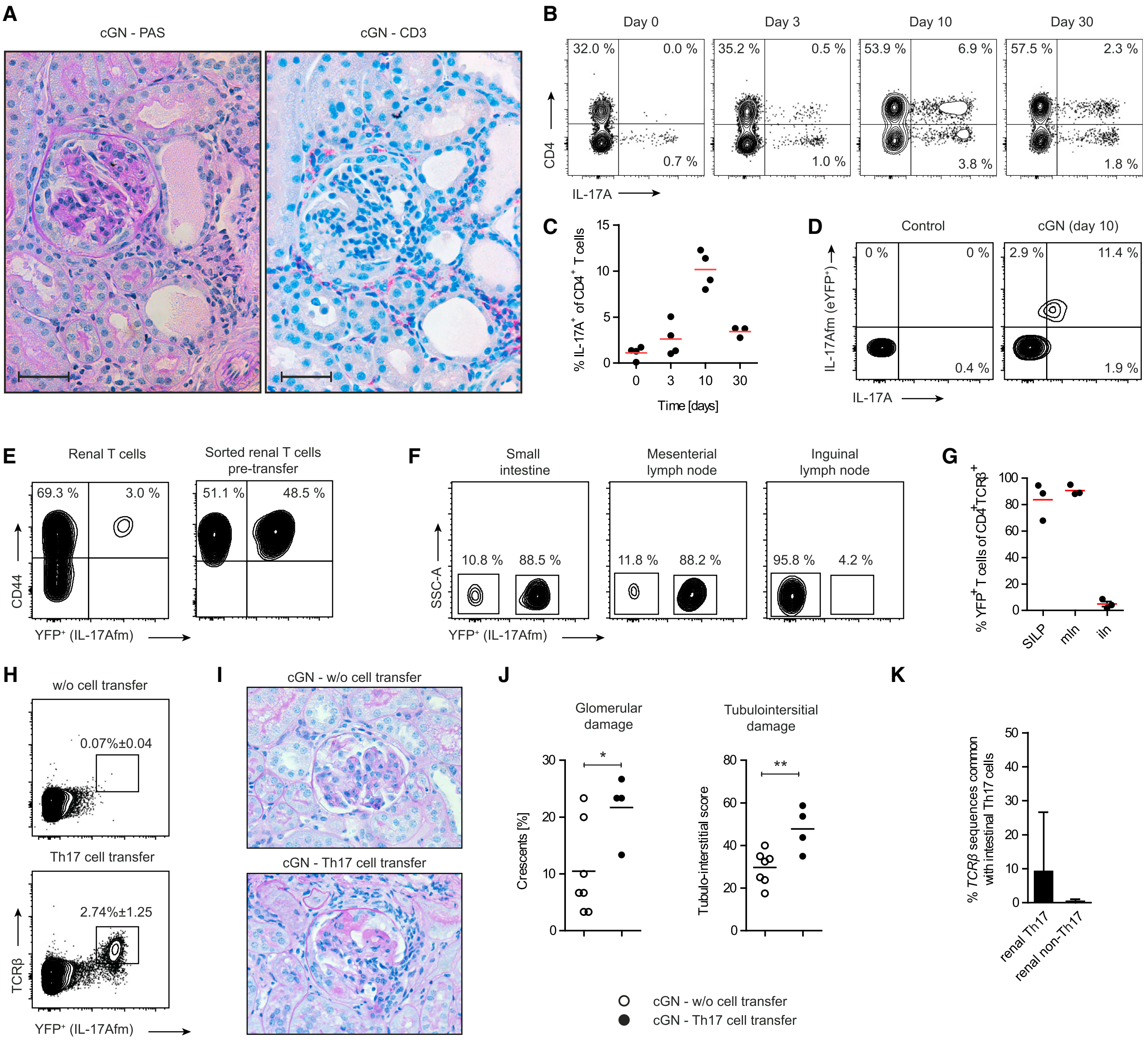

Figure 2. Renal Th17 Cells Have Gut-Homing Properties

(A) PAS and CD3 staining of renal tissue sections of mice at day 10 after induction of crescentic glomerulonephritis (cGN). Scale bar, 20 μm.

(B) Intracellular cytokine staining and FACS analyses of $CD3^+$ T cells in the course of cGN (day 0–30).

(C) Quantification of $IL\text{-}17A^+CD4^+$ Th17 cells in the course of cGN.

(D) Flow cytometry of renal $CD4^+$ T cells from *Il17a* fate reporter mice ($Il17a^{Cre}$ × $R26R^{eYFP}$) and intracellular staining for IL-17A expression.

(E) $CD44^{high}$ Th17 cells (YFP^+) and $CD44^{high}$ non-Th17 cells (YFP^-) were FACS sorted from the kidneys of *Il17a* fate reporter mice with cGN. Cells were mixed in a 1:1 ratio and transferred into $Tcr\alpha^{-/-}$ recipients.

(F and G) After 12 weeks, small intestinal lamina propria (SILP), mesenteric lymph nodes (mln), and inguinal lymph nodes (iln) were analyzed for the abundance of Th17 cells (YFP^+) and non-Th17 cells (YFP^-) by flow cytometry.

(H) Transfer of FACS-sorted Th17 cells ($CD44^{high}YFP^+$) from the small intestine of *Il17a* fate reporter mice into $Tcr\alpha^{-/-}$ recipients and cGN induction 5 weeks after cell transfer. At day 8 after cGN induction, the kidneys were analyzed for gut-derived Th17 cells.

(I and J) (I) PAS-stained kidney sections and (J) quantification of renal damage of mice after Th17 cell transfer and subsequent cGN induction.

(K) cGN was induced in *Il17a* fate reporter mice. At day 10, renal Th17 cells (YFP^+), renal non-Th17 cells (YFP^-), and small-intestinal Th17 cells were FACS sorted and analyzed for TCRβ sequences using the ImmunoSEQ platform. Common sequences of intestinal Th17 and renal Th17 or renal non-Th17 cells were calculated. Error bars indicate SEM.

See also Figure S2. Data are representative of at least two independent experiments. Symbols represent individual data points with the mean as a horizontal line, bars represent mean values. $^*p < 0.05$, $^{**}p < 0.01$.

than with renal non-Th17 cells. Thus, gut and kidney Th17 cells might be reactive to identical antigens. However, further studies are clearly needed to characterize the antigen specificity of renal and gut Th17 in cGN. This is currently hampered by the lack of well-characterized $CD4^+$ T cell epitopes in the cGN model.

Taken together, these data provide first evidence for the relationship of intestinal and renal Th17 cells and suggest the potential migration of Th17 cells from the small intestine into the kidney in crescentic glomerulonephritis.

Th17 Cells in Glomerulonephritis Migrate from the Intestine into the Kidney

To investigate the potential migration of T cells from the small intestine into the kidney in glomerulonephritis, we used mice engineered to ubiquitously express Kaede (Tomura et al., 2010). Kaede is a photoconvertible protein, which permanently changes its fluorescence emission from green (518 nm) to red (582 nm) upon photoactivation with near-UV light (350–410 nm). After selective exposure of the small intestine for 60 s, Kaede-photoconversion was specific to cells in the small intestine (Figure S3).

Next, we induced cGN in *Kaede*-transgenic mice and photoconverted intestinal cells at day four (Figure 3A). At day seven, confocal microscopy of kidney sections revealed the presence of Kaede red^+ cells in the tubulointerstitial area (Figure 3B). Furthermore, migration of Kaede red^+ cells into the inflamed kidney could be detected by flow cytometry, whereas under non-nephritic conditions, no significant migration of Kaede red^+ cells was present (Figures 3C and 3D). Besides, intravascular staining using an anti-CD45 antibody 3 min before nephrectomy allowed the discrimination between tissue-localized and intravascular blood cells (Anderson et al., 2014) and demonstrated that Kaede red^+ cell were indeed located within the inflamed kidney (Figure S3), ruling out a contamination by circulating blood cells.

Most importantly, the percentage of IL-17A-positive cells was significantly higher in gut-derived Kaede red^+ cells as compared to Kaede $green^+$ cells (Figures 3C and 3D), demonstrating the preferential migration of Th17 cells from the intestine into the inflamed kidney. In contrast, IFN-γ-expressing Th1 cells were underrepresented in renal Kaede red^+ cells (Figure 3D). There was also no accumulation of IL-17A-producing $\gamma\delta$ T cells among Kaede red^+ cells (Figures 3E and 3F), supporting the idea that $\gamma\delta$ T cells reside in the target organ.

Th17 Cell Egress from the Small Intestine Is Dependent on S1P Receptor 1

The trafficking of Th17 cells from the intestine into the kidney requires the egress from the small intestinal lamina propria into the lymphatics. The mechanisms of T cell egress from extralymphoid tissue, in particular under inflammatory conditions, are poorly defined. It has been suggested that potential "exit receptors," such as CCR7 and the S1P receptor 1, might promote T cell egress, whereas "retention signals," e.g., CD103, might exert the opposite effect.

Flow cytometry of $eYFP^+$ Th17 cells from the small intestine of nephritic *Il17a*^{Cre} × *R26R*^{eYFP} mice showed almost uniform surface expression of the activation marker CD44 and a high level of CD69 and CCR6 expression, whereas CD103 and CCR7 were hardly detectable (Figures 4A–4C). Due to the lack of suitable FACS antibodies for the S1P receptor 1, we sorted Th17 cells from the small intestine of nephritic and non-nephritic *Il17a* fate reporter mice and performed RT-PCR analysis. Interestingly, intestinal Th17 cells upregulated the mRNA expression of S1P receptor 1 and its major transcription factor KLF2 under nephritic conditions (Figure 4D), suggesting a potential function of this receptor for the egress of Th17 cells from the gut.

Consequently, we induced cGN in *Kaede*-transgenic mice, photoconverted intestinal cells at day four, and treated these mice from day four to seven with FTY720 (Figure 4E), a functional S1P receptor 1 agonist that arrests lymphocyte trafficking from lymphoid organs into systemic circulation. FTY720 treatment blocked the exit of $CD4^+$ T cells from the small intestine (Figures 4F and 4G). Accordingly, the trafficking of Kaede red $CD4^+$ T cells, including Th17 cells, from the intestine into the mesenteric lymph node (Figure 4H) and subsequently into the kidney was significantly reduced (Figures 4I–4K). In contrast, by using *Kaede Ccr7*$^{-/-}$ mice, we demonstrated that the lack of *Ccr7* affected neither the emigration of $CD4^+$ T cells out of the gut nor the migration of Th17 cells into the kidney (Figures 4F–4K). Taken together, these experiments revealed that Th17 cell egress from the small intestine is dependent on S1P receptor 1.

Of note, additional S1P receptor 1 blocking experiments demonstrated that the FTY720 application significantly reduced renal Th17 cell infiltration and subsequent kidney pathology in nephritic mice (Figures S4A–S4D). These results further support—but due to the pleiotropic effect of FTY720, do not definitively confirm—the therapeutic potential of blocking Th17-cell gut egress in cGN.

CCR6/CCL20 Axis Guides the Trafficking of Intestinal Th17 into the Inflamed Kidney

Following the exit from the gut, Th17 cells have to migrate via the circulation into the kidney. Th17 cells in the kidney highly express CCR6 (Figures 4L and 4M), which is accompanied by an upregulated expression of its unique ligand CCL20 in the inflamed kidney (Figure 4M). To find out whether the CCR6/CCL20 axis regulates the trafficking of intestinal Th17 into the kidney, nephritic *Kaede* mice were treated either with a neutralizing anti-CCL20 antibody or an isotype control antibody. The trafficking of photoconverted Th17 cells from the gut into the nephritic kidney was significantly reduced in mice treated with the anti-CCL20 antibody (Figures 4N and 4O). In contrast, Th1 cell recruitment was not affected (Figure 4N). Of note, anti-CCL20 treatment did not affect the emigration of $CD4^+$ Th17 cells from the small intestine (data not shown). Moreover, CCL20 neutralization did not influence the clinical course of cGN (Figures S4E–S4G). This is in line with a recent study demonstrating that the CCL20/CCR6 axis also mediates renal recruitment of Tregs, and that the reduction of anti-inflammatory Tregs in the presence of a fully functional Th1 response aggravates experimental glomerulonephritis (Turner et al., 2010).

Renal Th17 Cell Responses and Tissue Injury in cGN Is Attenuated in Germ-Free Mice

To evaluate the functional impact of intestinal Th17 cells on the course of Th17-driven experimental cGN, we induced glomerulonephritis in C57BL/6 mice raised and kept under germ-free (GF) or specific pathogen-free (SPF) conditions. The absence of microbiota in GF mice (GFM) resulted in a deficiency

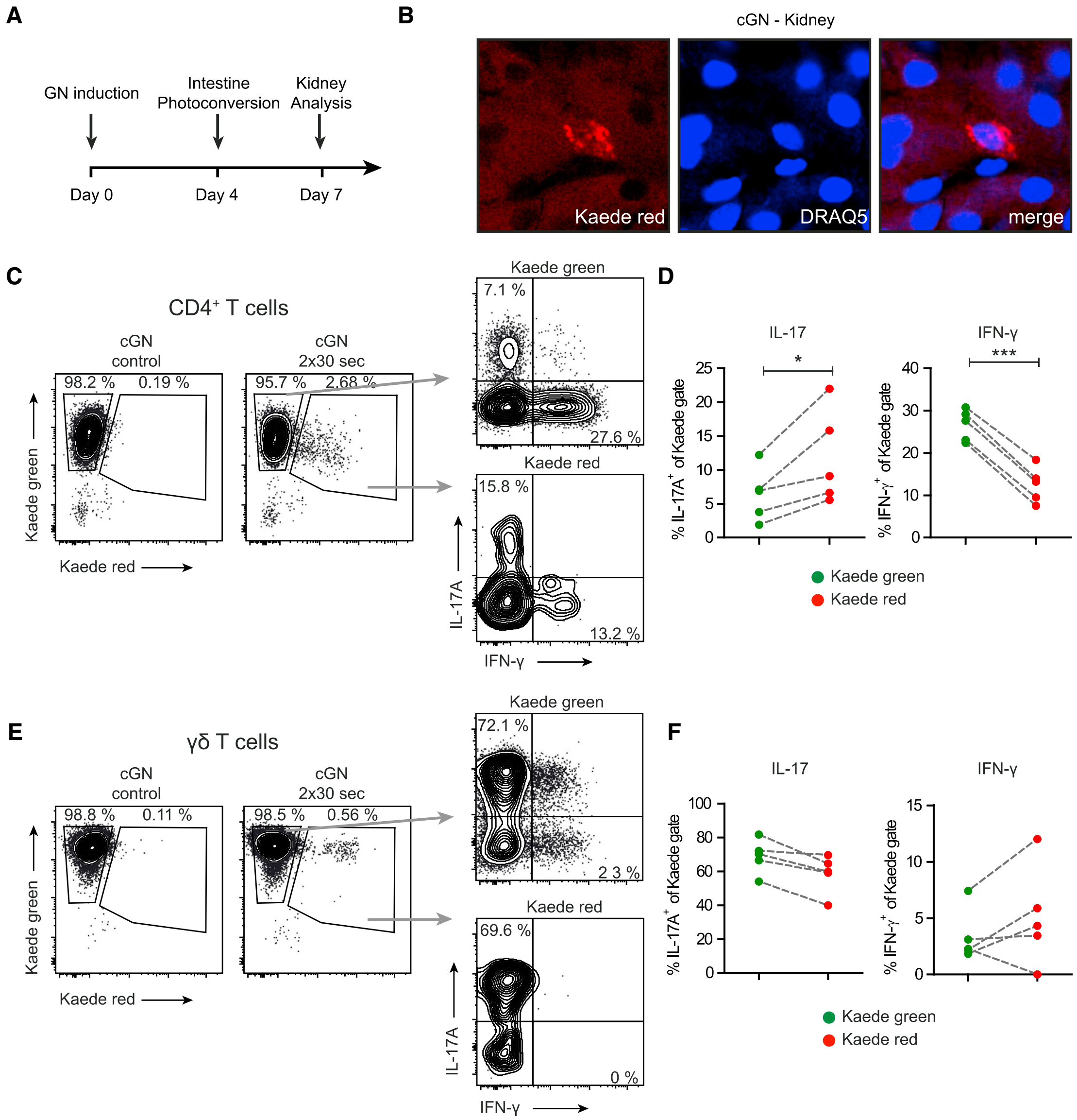

Figure 3. Th17 Cells in Glomerulonephritis Migrate from the Intestine into the Kidney

(A) Experimental procedure: 4 days after cGN induction, photoconversion of the small intestine was performed, and at day 7, the kidneys were analyzed.

(B) Confocal microscopy of Kaede red+ cells in a renal tissue section of *Kaede*-tg mice (nucleus: DRAQ5-staining, blue).

(C) Flow cytometry of renal CD4+ T cells from *Kaede*-tg mice after cGN induction and photoconversion of the small intestine and controls. Kaede green+ cells and Kaede red+ cells were assessed for IL-17A and IFN-γ expression.

(D) Quantification of IL-17A and IFN-γ expression in the respective Kaede population.

(E and F) Analysis and quantification of IL-17A and IFN-γ expression in Kaede green+ and Kaede red+ γδ T cells. Data are representative of three independent experiments. Symbols represent individual data points, with the mean as a horizontal line.

*$p < 0.05$, ***$p < 0.001$. See also Figure S3.

of intestinal Th17 cells (Figure 5A). Ten days after GN induction, Th17 cells were reduced in the kidneys of nephritic GFM, while the percentage of Th1 cells was unchanged (Figures 5B and 5C). In line, PAS-stained kidney sections revealed less glomerular and tubular damage in nephritic GFM compared to SPF mice (Figures 5D and 5E).

In contrast to the ameliorated course of GN in GFM, there was no difference in renal Th17 responses (Figures S5A and S5B) or renal tissue injury between nephritic mice and conventionally colonized ex-GFM (Figure S5C).

Depletion of the Gut Microbiota by Broad-Spectrum Antibiotics Ameliorates Th17 Cell Responses in cGN

To investigate whether the manipulation of gut microbiota in SPF mice might prevent Th17-promoted kidney damage, we orally treated mice with a cocktail of four antibiotics (ampicillin,

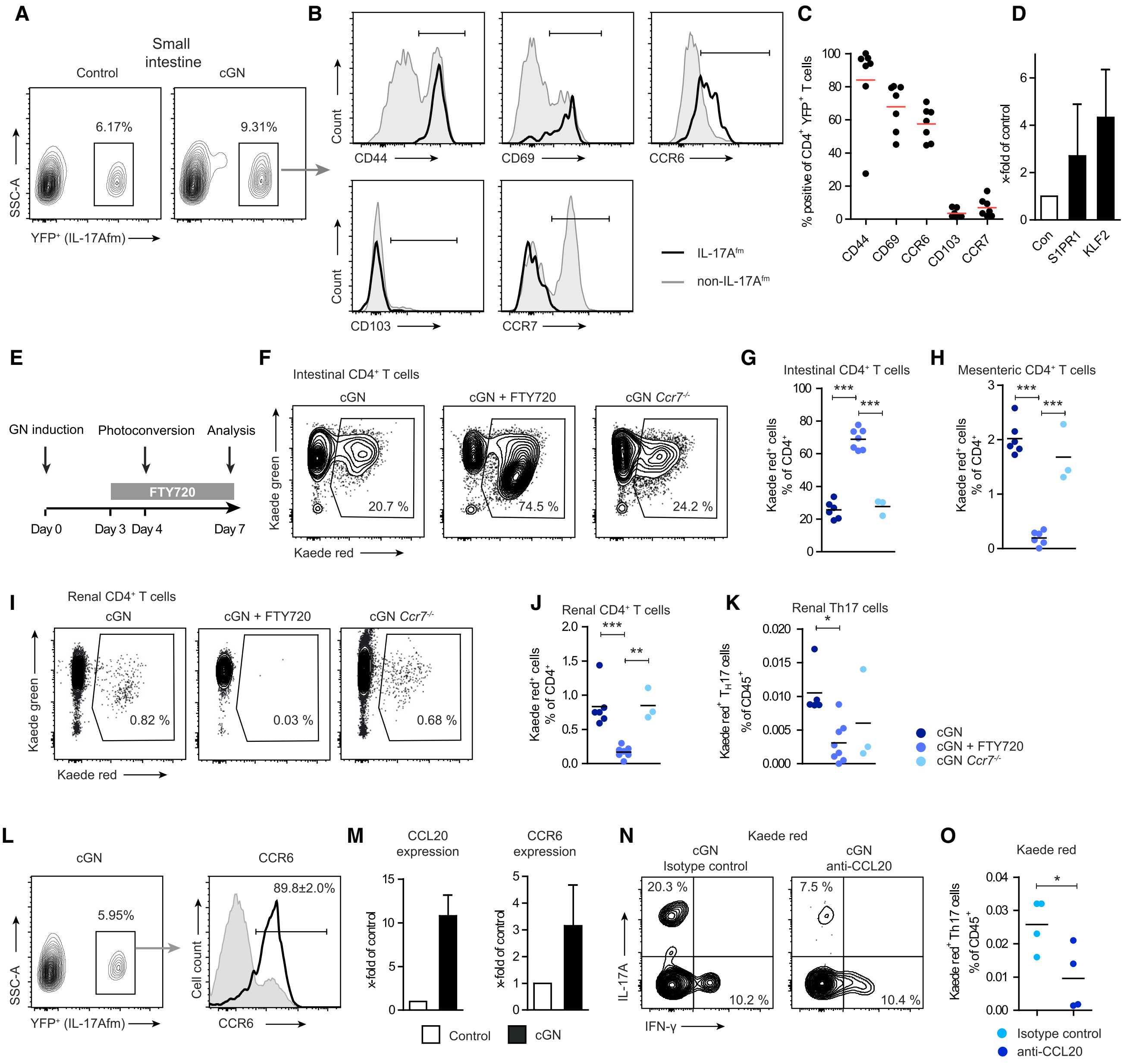

Figure 4. Th17 Cells Egress from the Intestine in a S1P-Receptor-1-Dependent Fashion and Migrate to the Kidney via the CCL20/CCR6 Axis in cGN

(A–C) Flow cytometry of T cells from the small intestinal lamina propria of *Il17a* fate reporter mice for CD44, CD69, CCR6, CD103, and CCR7 as indicated.

(D) RT-PCR analysis of FACS-sorting $CD4^+$ Th17 cells from the small intestinal lamina propria of *Il17a* fate reporter mice. Error bars indicate SEM.

(E) After cGN induction in *Kaede* control (±FTY720 treatment day 3–7) and *Kaede Ccr7*$^{-/-}$ mice, intestinal cells were photoconverted and subsequently analyzed for Kaede red^+ $CD4^+$ T cells.

(F–K) FACS analysis of intestinal (F and G), mesenteric lymph node (H), and renal (I–K) $CD4^+$ T cells. See also Figures S4A–S4D.

(L) Renal Th17 cells from *Il17a* fate reporter mice at day 10 after cGN induction were analyzed for CCR6 expression by flow cytometry.

(M) Quantitative RT-PCR of CCL20 and CCR6 in the kidneys of mice after induction of cGN and control mice (n = 4 per group). Error bars indicate SEM.

(N and O) cGN was induced in *Kaede*-tg mice. From day 4 until 7, these mice received anti-CCL20 monoclonal antibody or isotype control. Photoconversion was performed at day 4. At day 7, renal $CD4^+$ Kaede red^+ T cells were analyzed for IL-17A and IFN-γ expression.

See also Figure S4E–S4G. Data are representative of three independent experiments. Symbols represent individual data points, with the mean as a horizontal line, bars represent mean values. *$p < 0.05$, **$p < 0.01$, ***$p < 0.001$.

metronidazole, neomycin, and vancomycin [AMNV]) prior cGN induction. In line with a recent report (Horai et al., 2015), AMNV treatment almost depleted the gut microbiota (Figure S6) and resulted in a reduced accumulation of Th17 cells in the small intestine (Figure 5F). Even more important, the number of renal Th17 cells (Figures 5G and 5H), but not Th1 cells, and the subsequent

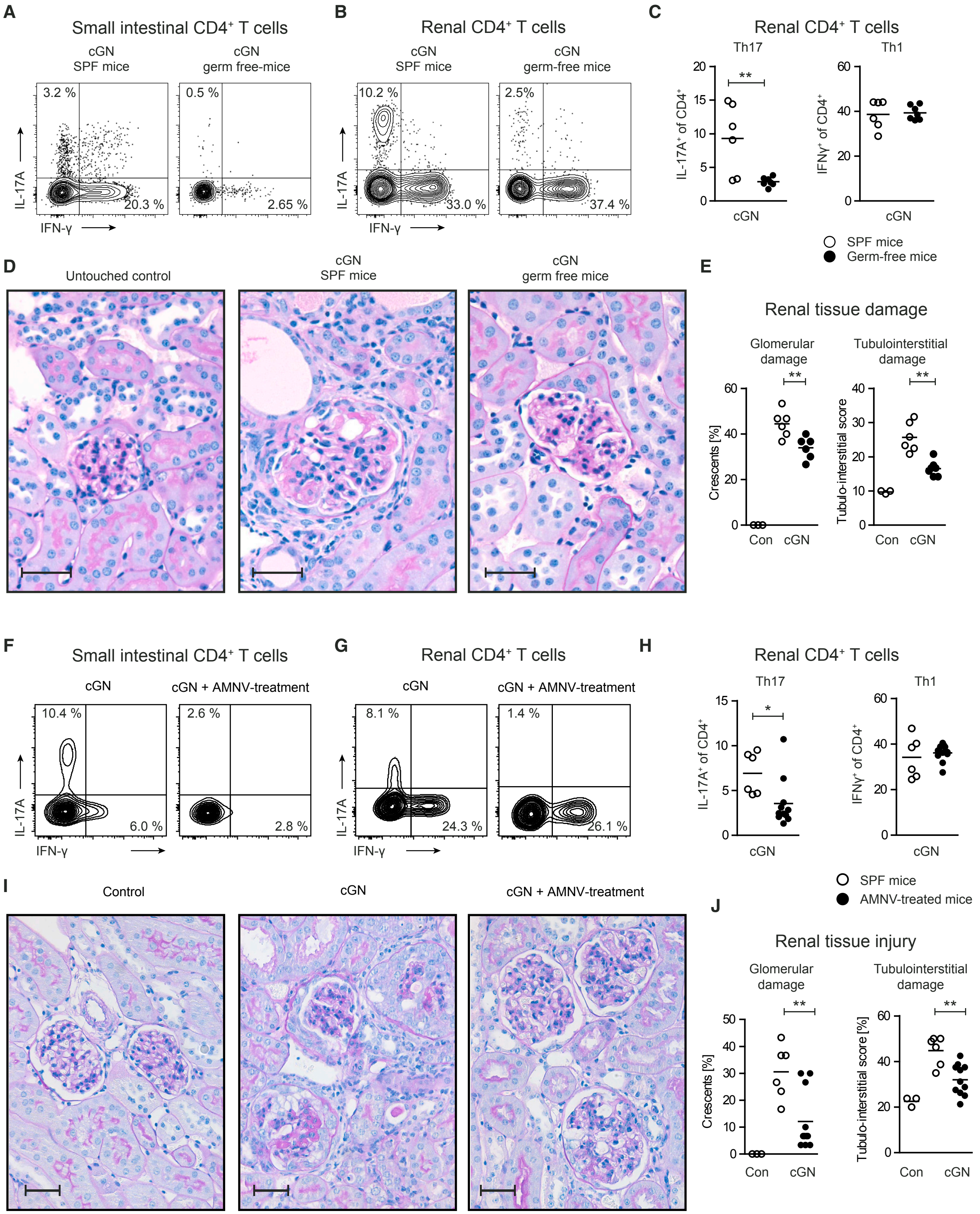

Figure 5. Renal Th17 Response in cGN Is Dependent on Intestinal Microbiota

(A) Flow cytometry of CD4+ T cells from the small intestinal lamina propria of GFM and SPF mice after induction of cGN.

(B and C) Flow cytometry of renal CD4+ T cells for IL-17A and IFN-γ expression.

(legend continued on next page)

glomerular and tubulointerstitial injury were reduced (Figures 5I and 5J).

Expansion of Intestinal Th17 Cells in *Citrobacter-Rodentium*-Infected Mice Promotes Renal Th17 Responses in cGN

Next, we infected nephritic mice at day 0 with *Citrobacter rodentium* that triggers a potent Th17 cell response in the gut (predominantly in the colon and, to a lesser degree, in the small intestine) seven days after oral challenge (Figure 6A) (Collins et al., 2014). Flow-cytometric analysis revealed that, indeed, the Th17 response was markedly upregulated in the kidneys of nephritic *C.-rodentium*-infected mice, while renal Th1 response and IL-17A production by $\gamma\delta$ T cells were unchanged (Figures 6B and 6C). In accordance with the enhanced Th17 response, the recruitment of neutrophils into the kidney was increased (Figure 6D). Moreover, *C.-rodentium*-infected mice developed a moderately aggravated course of nephritis in terms of glomerular crescent formation and tubulointerstitial injury (Figure 6E).

Therapeutic Manipulation of the Gut Microbiota with Vancomycin Reduces Th17-Cell-Driven Injury in cGN

Finally, for a more specific therapeutic targeting of microbiota-induced Th17 cells in the gut, we treated mice orally with the glycopeptide antibiotic vancomycin (starting 4 weeks before GN induction). Vancomycin is not absorbed in the intestine, thus preventing major systemic side effects, and predominantly targets gram-positive bacteria, including Clostridia species. To analyze intestinal microbiota composition after vancomycin treatment, we applied amplicon sequencing. As expected, vancomycin treatment, in contrast to a combination of four antibiotics, which essentially eradicates the whole gut microbiome (Figure S6), reduced the diversity of the intestinal microbiota without depleting all commensal bacteria (Figures 7A and 7B). In particular, the family Clostridiales (phylum Firmicutes), which has been previously shown to promote intestinal Th17 cells (Ivanov et al., 2009), was reduced by this treatment. Furthermore, increased abundance of Enterobacteriaceae, Lactobacillaceae, and Verrucomicrobiaceae were detected (Figure 7B). Of note, segmented filamentous bacteria were not present.

Treatment with vancomycin was sufficient to selectively decrease the number of Th17 cells in the small intestine (Figures 7C and 7D). As seen in nephritic GFM and nephritic mice treated with broad-spectrum antibiotics, the diminished numbers of Th17 cells in the gut resulted in a reduced recruitment of Th17 cells, but not Th1 cells, into the kidney (Figure 7E). Vancomycin-treatment also reduced IL-17A expression by intestinal $\gamma\delta$ T cells (Figures S7A and S7B) but did not affect IL-17A expression by renal $\gamma\delta$ T cells in cGN (Figures S7C and S7D). Accordingly, renal mRNA expression analysis, using a cytokine- and chemokine-pathway-focused PCR array (RT^2 Profiler), revealed a predominant downregulation of Th17/IL-17A target genes in vancomycin-treated mice (Figure S7E). Subsequent RT-PCR analysis confirmed the reduced mRNA expression of the Th17 pathway, including the neutrophil chemoattractants CXCL1 and CXCL5 (Figure S7F). In line, we observed decreased neutrophil recruitment into the kidney (Figures 7F and 7G). No major effect of vancomycin on the humoral immune response to the nephritogenic antigen was detectable (Figures S7G–S7I). Vancomycin-treated animals developed less severe disease in terms of renal tissue injury (Figures 7H and 7I) and a better-preserved kidney function as measured by blood urea nitrogen (BUN) and albumin-to-creatinine ratio (ACR) (Figure 7J), highlighting the therapeutic potential of this approach. Of note, vancomycin application in nephritic *Il17a*$^{-/-}$ mice had no effect on renal tissue injury or neutrophil recruitment (Figures 7K–7M), indicating that the ameliorated course of the disease in vancomycin-treated mice is indeed Th17/IL-17A-dependent.

DISCUSSION

Rapidly progressive or cGN is the most aggressive form of autoimmune kidney disease and remains a significant cause of end-stage renal failure. Different disease entities may lead to the development of cGN. The most common cause is ANCA-associated small vessel vasculitis (Couser, 2012; Kurts et al., 2013). Although there has been some progress in the treatment of patients with cGN, the use of unspecific immunosuppressive or cytotoxic agents is still recommended in guidelines, illustrating the fact that more specific treatment options based on the underlying immunopathogenic mechanisms are clearly needed.

There is convincing evidence for a pathogenic role of Th17 cells in murine models of crescentic and proliferative GN (Gan et al., 2010; Hünemörder et al., 2015; Krebs et al., 2013; Ooi et al., 2009; Paust et al., 2012; Paust et al., 2009; Pisitkun et al., 2012; Ramani et al., 2014; Steinmetz et al., 2011; Tulone et al., 2011), but the translation of these findings into new therapeutic approaches has been hindered by the lack of robust data about the role of the Th17 immune response in patients with cGN. Our flow cytometric analysis revealed that CD4$^+$RORγt$^+$ Th17 cell frequencies were up to 30% in the kidney of ANCA-GN patients, which is higher than the reported frequencies in most other tissues affected by autoimmune diseases (Annunziato et al., 2013). The local Th17 cell response promotes kidney injury by recruiting neutrophils and other leukocyte subtypes to the target tissue in mice (Disteldorf et al., 2015), and its presence in human patients is a prerequisite for translating the findings gained from animal models into clinical practice, for example, as successfully done in IL-17A targeting for the treatment of psoriasis (Leonardi et al., 2012; Mease et al., 2014).

(D) PAS staining of renal cortex from cGN and control mice. Scale bar, 20 μm.
(E) Glomerular crescent formation and tubulointerstitial damage (score) in the respective groups. See also Figure S5.
(F–J) Mice were treated with a combination of 4 antibiotics (AMNV) for 4 weeks prior to induction of cGN. (F) Small intestinal CD4$^+$ T cells and (G) renal CD4$^+$ T cells were analyzed for IL-17A and IFN-γ. (H) Quantification of cytokine expression in renal CD4$^+$ T cells. (I) PAS staining of renal cortex of mice with AMNV treatment and control mice. (J) Quantification of renal damage.
See also Figure S6. Data are representative of three independent experiments. Symbols represent individual data points, with the mean as a horizontal line. Scale bar, 20 μm. **p < 0.01.

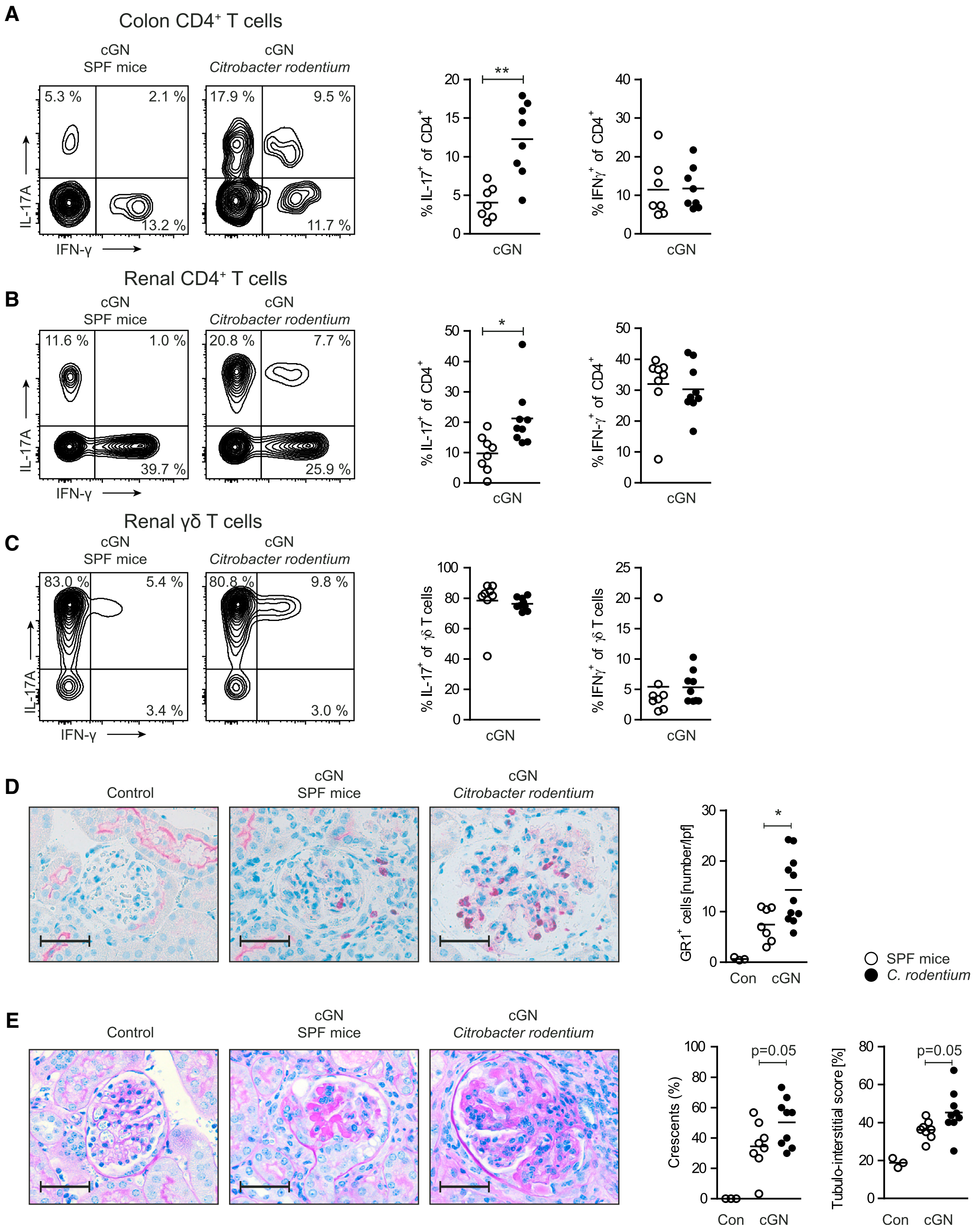

Figure 6. Expansion of Intestinal Th17 Cells by *Citrobacter Rodentium* Infection Aggravates Renal Th17 Immune Responses in cGN

(A) Flow cytometry of large intestine lamina propria CD4+ T cells after infection with *Citrobacter rodentium* and induction of cGN.

(B and C) (B) Renal CD4+ T cells and (C) renal γδ T cells were analyzed for intracellular cytokine expression.

(D) GR-1 staining and quantification of neutrophil infiltration in the renal cortex from mice with cGN.

(E) Kidney damage was assessed by evaluation for glomerular crescents and tubulointerstitial damage in PAS-stained renal cortex sections. Scale bar, 25 μm. Data are representative of three independent experiments. Symbols represent individual data points, with the mean as a horizontal line.*$p < 0.05$, **$p < 0.01$.

The developmental origin of Th17 cells, promoting organ-specific autoimmunity, remains largely unexplored. Under homeostatic conditions, Th17 cells are most abundant in the gut, where their induction and accumulation depends on the gut microbiota (Atarashi et al., 2015; Ivanov et al., 2008, 2009; Sano et al., 2015). Moreover, the induction of intestinal Th17 cells by commensal

microbes, e.g., SFB, and the lack of Th17 cells in GFM, have profound effects on extra-intestinal autoimmune disorders (Lee et al., 2011; Wu et al., 2010). This suggests a direct functional relationship between microbiota-induced Th17 cells in the gut and Th17-driven tissue injury at peripheral sites, e.g., the kidney.

To determine whether renal Th17 cells might be derived from the gut, we induced the Th17-cell-dependent model of cGN (Bollée et al., 2011; Krebs et al., 2013; Pisitkun et al., 2012; Tsuboi et al., 2008) in photoconvertible *Kaede*-transgenic mice (Tomura et al., 2010). After photoconversion of cells in the small intestine, we were able to detect a significant proportion of gut-derived Th17 cells in the inflamed kidneys. Of note, due to technical limitations of the Kaede system that cannot be overcome at present (e.g., photoconversion in the small intestine was not > 75%, did not include all segments of the gut, and covered only a short period of time), our finding did not provide final evidence that glomerulonephritis-driving Th17 cells are exclusively or predominantly derived from the gut. Using a related technical approach, Morton et al. (2014) demonstrate the movement of Th17 cells from the ascending colon into the spleen in arthritis-prone K/BxN mice, and Mackley et al. (2015) reveal a constitutive trafficking of RORγt$^+$ ILC3s from the intestine to the draining mesenteric lymph nodes. Recently, Benakis et al. (2016) demonstrate that antibiotic-induced alterations in the intestinal flora reduce ischemic brain injury in mice, potentially as a consequence of a reduction in meningeal IL-17-positive $\gamma\delta$ T cells. The authors use photoconvertible KiK mice to track cells from the gut, but they do not provide direct evidence for the migration of IL-17-producing cells ($\gamma\delta$ T cells or CD4$^+$ T cells) from the intestine to the CNS. In line with this report, we showed that targeting of the intestinal microbiota by vancomycin not only reduced IL-17-producing CD4$^+$ T cells but also IL-17A-producing $\gamma\delta$ T cells in the gut. However, in contrast to the study by Benakis et al. (2016), these interventions did not influence IL-17A-producing $\gamma\delta$ T cells at extra-intestinal sites, namely the inflamed kidney, supporting the idea that $\gamma\delta$ T cells in cGN predominantly reside in the kidney and are not derived from the gut.

Our data provide direct evidence for the trafficking of Th17 cells from the gut to extra-intestinal sites of Th17-driven inflammation. The trafficking of Th17 cells from the intestine into the inflamed kidney in cGN required the exit from the small intestinal lamina propria into the lymphatics and, subsequently, via the circulation into the kidney. Effector T cells use S1P receptors to sense S1P gradients among blood, tissues, and lymph, thereby guiding entry into efferent lymphatics during egress from lymphoid tissues (Baeyens et al., 2015); however, whether this concept also applies for non-lymphoid organs, e.g., the intestine, is less well characterized. Here, we found that Th17 cell egress from the small intestine was dependent on S1P receptor 1 and that their subsequent trafficking into the inflamed kidney was mediated via the CCL20/CCR6 axis.

Whether Th17 cells, generated in the intestine in response to microbes, represent a general "reservoir" for Th17 cells, which can be mobilized and migrate to distant sites of inflammation in autoimmune or infectious diseases, remains to be fully elucidated. Furthermore, it would be of great interest to study whether circulating Th17 cells from the gut are recruited primarily into the nephritic kidney via local chemoattractants or whether, in addition, so-far-unidentified kidney-derived signals might mobilize Th17 cells to exit the gut.

The absence of intestinal Th17 cells in GFM and their depletion in mice treated with broad-spectrum antibiotics resulted in a reduced renal Th17 response and ameliorated the consecutive tissue injury in glomerulonephritis. In contrast, expansion of intestinal Th17 cells in *Citrobacter-rodentium*-infected nephritic mice exerted the opposite effect. Most importantly, further experiments revealed that oral application of vancomycin alone was sufficient to reduce microbiota-induced intestinal Th17 cells and Th17 responses in the kidney, resulting in an ameliorated course of cGN without any significant side effects, emphasizing the great potential of this novel treatment strategy.

Before the manipulation of gut microbiota with, for example, antibiotics is tested as a therapeutic strategy in Th17-cell-driven human autoimmune disorders, a better understanding of the interaction of the microbiome and Th17 cells in the human intestine is clearly needed. But the finding that treatment with co-trimoxazole, given twice daily for 24 months, prevented relapses in patients with ANCA-associated vasculitis, in particular with upper respiratory disease (Stegeman et al., 1996), is of interest. The mechanisms by which co-trimoxazole acts are still elusive, but it suggests a possible therapeutic role for antimicrobial therapy. Because this study was performed long before the first identification of Th17 cells, the effect of co-trimoxazole on gut microbiota and Th17 responses was not assessed.

EXPERIMENTAL PROCEDURES

Animals

Kaede-transgenic mice were obtained from M. Tomura (Kyoto University) (Tomura et al., 2010). *Il17a*$^{-/-}$ mice were provided by Y. Iwakura (University of Tokyo). *Ccr7*$^{-/-}$ and *Tcr*$\alpha^{-/-}$ mice were from The Jackson Laboratory. *Il17a*^{CRE} × *R26R*^{eYFP} mice have previously been described (Hirota et al., 2011). Mice were on the C57BL/6J background and raised in SPF conditions. GFM were raised and kept under sterile conditions (Steinhoff et al., 1999). All animal experiments were approved by the local committees.

Animal Procedures

Experimental cGN was induced by i.p. injection of nephrotoxic sheep serum in 8- to 12-week-old male mice (Bollée et al., 2011; Krebs et al., 2013; Pisitkun et al., 2012; Tsuboi et al., 2008). For photoconversion, the small intestine of anesthetized *Kaede*-transgenic mice was subjected to lighting using a Blue Wave LED Prime UVA (Dymax). For urine analyses, mice were housed in metabolic cages for 5 hr, and urinary albumin was determined by ELISA (Bethyl Laboratories). For *Citrobacter rodentium* infections, mice were inoculated with 200 μL of a bacterial suspension (10^9 CFU/mouse) via an oral gavage (Nagai et al., 2005).

Interventional Studies

Mice were given either a combination of antibiotics (ampicillin 1 g/L, metronidazole 1 g/L, neomycin 1 g/L, and vancomycin 0.5 g/L) or vancomycin alone (0.5 g/L) by drinking water 4 weeks prior to the induction of cGN (Ivanov et al., 2008; Rakoff-Nahoum et al., 2004). Anti-CCL20-antibody (clone 114908, R&D Systems) or isotype control (clone 43414, R&D Systems) were used at 50 μg/day per mouse (i.p.) at days 4, 5, and 6 after cGN induction (Kallal et al., 2010). FTY720 was added to the drinking water at 5 μg/mL (Kursar et al., 2008). FTY720 treatment was initiated 3 days after cGN induction and maintained until analysis.

Real-Time PCR Analyses

Total RNA of the renal cortex was prepared according to standard laboratory methods. Real-time PCR was performed for 40 cycles on a StepOnePlus

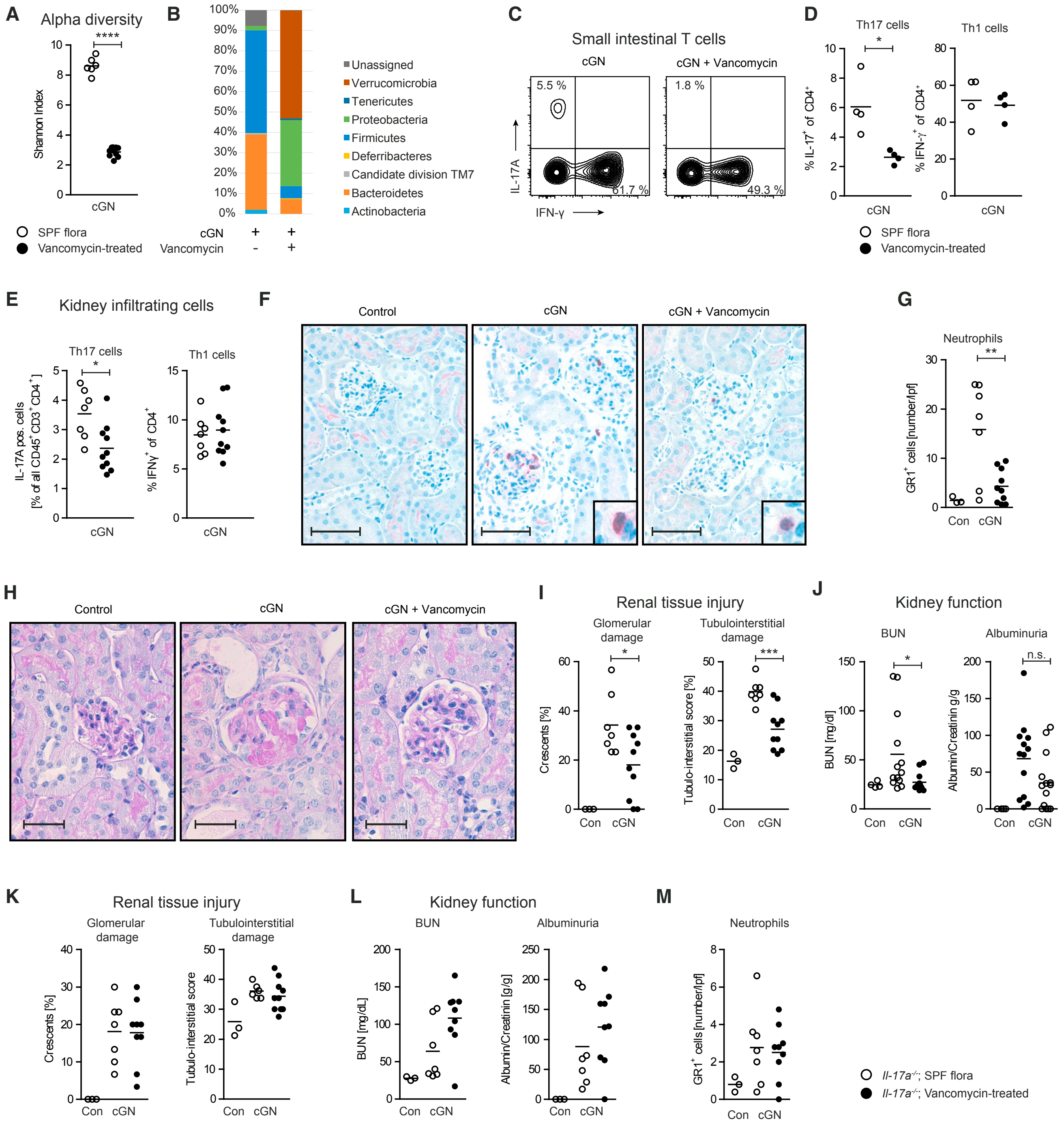

Figure 7. Manipulation of the Gut Microbiota with Vancomycin Ameliorates Th17-Cell-Driven Injury in cGN

Mice were treated for 4 weeks with vancomycin via the drinking water prior to induction of cGN. At day 10, mice were sacrificed, and stool samples were analyzed by next-generation sequencing for microbiota abundance.

(A) Alpha diversity of microbiota abundance in mice with cGN, with and without vancomycin treatment.

(B) Abundance of bacteria on phylum level in mice after vancomycin treatment and control.

(C) Intestinal CD4$^+$ T cells from mice with and without vancomycin treatment were analyzed for cytokine production.

(D and E) Quantification of IL-17A and IFN-γ in (D) intestinal CD4$^+$ T cells and (E) renal CD4$^+$ T cells after vancomycin treatment.

(F and G) (F) GR-1 staining (scale bar, 50 μm) and (G) quantification of renal neutrophil infiltration.

(H and I) (H) PAS staining (scale bar, 25 μm) and (I) quantification of renal tissue damage in mice after vancomycin treatment and induction of cGN.

(J) BUN and albuminuria as functional parameters for kidney damage were measured in the respective groups. cGN induction was also assessed in vancomycin-treated *Il17a*$^{-/-}$ mice.

(legend continued on next page)

Real-Time PCR system (Applied Biosystems) as previously described (Krebs et al., 2013). All samples were run in duplicate and normalized to 18S rRNA.

Morphological Analyses
Glomerular injury and crescent formation, deposition of PAS-positive material, and tubulointerstitial injury were assessed in PAS-stained renal tissue sections (Krebs et al., 2013). Further morphological analyses are described in Supplemental Experimental Procedures.

Leukocyte Isolation and Transfer
For T cell transfer experiments, $CD4^{+}$ T cells were isolated from kidneys of *Il17a*^{Cre} × *R26*^{eYFP} fate reporter mice at day 10 after induction of cGN or from the small intestine of these mice without cGN. Cells were sorted on a FACS Aria IIIa system (Krebs et al., 2013).

Flow Cytometry
Measurements were performed on a BD FACS LSR II or a BD LSR II Fortessa (BD Biosciences), and data were analyzed with the FlowJo (Tree Star). Cells were stained with antibodies from Biolegend, BD Biosciences, and eBioscience. LIVE/DEAD staining (Thermo Fisher) was used to exclude dead cells. For intracellular cytokine staining, cells were fixed and permeabilized using the Cytofix/Cytoperm kit (BD Bioscience) or 3.7% PFA/0.1% Igepal in the case of reporter mice (Hirota et al., 2011).

Analyses in Patients with ANCA-GN
Single-cell suspensions were obtained from human biopsies by enzymatic digestion followed by dissociation with gentleMACS (Miltenyi Biotec), antibody staining, and flow cytometry (Paust et al., 2015). Analyses of human kidney biopsies were approved by the local ethics committees (PV3162).

Sequencing Analysis
TCR sequencing of FACS-sorted cells from *Il17a* fate reporter mice (*Il17a*^{Cre} × *R26R*^{eYFP}) and 16S rRNA sequencing of murine feces is described in Supplemental Experimental Procedures.

Statistical Analysis
Statistical analysis was performed using GraphPad Prism (La Jolla). The results are shown as the mean ± SEM when presented as a bar graph or as single data points with the mean in a scatter dot plot. Differences between two individual groups were compared using a two-tailed t test. In the case of three or more groups, a one-way ANOVA with Bonferroni's multiple comparisons test was used.

ACCESSION NUMBERS

All 16S rRNA sequencing data are submitted to EMBL-EBI with the accession number ENA: PRJEB15416.

SUPPLEMENTAL INFORMATION

Supplemental Information includes Supplemental Experimental Procedures and seven figures and can be found with this article online at http://dx.doi.org/10.1016/j.immuni.2016.10.020.

AUTHORS CONTRIBUTIONS

C.F.K., H.-J.P., S.K., T.K., U.S., and U.P. planned and performed experiments and analyses. C.F.K., J.-E.T., and U.P. designed the study and planned, as well as supervised, the research. C.F.K. and U.P. wrote the manuscript. S.R.B., J.-H.R., P. Bartsch, T.W., N.F., J.H., P. Busch, H.-W.M., B.S., R.A.K.S., C.M.-S., U.O.W., L.G.P., M.J., O.M.S., N.G., and S.H. performed experiments. C.F.K., J.-E.T., N.F., and U.P. analyzed the data and edited the manuscript.

ACKNOWLEDGMENTS

This study was supported by grants from the Deutsche Forschungsgemeinschaft (SFB 1192 to U.P. and C.F.K.) and grants from the Deutsche Nierenstiftung and Deutsche Gesellschaft für Nephrologie to C.F.K. T.K. received a scholarship from the Werner-Otto-Foundation. FACS sorting was performed at the UKE FACS sorting core facility.

Received: March 29, 2016
Revised: July 11, 2016
Accepted: September 27, 2016
Published: November 15, 2016

REFERENCES

Acosta-Rodriguez, E.V., Rivino, L., Geginat, J., Jarrossay, D., Gattorno, M., Lanzavecchia, A., Sallusto, F., and Napolitani, G. (2007). Surface phenotype and antigenic specificity of human interleukin 17-producing T helper memory cells. Nat. Immunol. *8*, 639–646.

Anderson, K.G., Mayer-Barber, K., Sung, H., Beura, L., James, B.R., Taylor, J.J., Qunaj, L., Griffith, T.S., Vezys, V., Barber, D.L., and Masopust, D. (2014). Intravascular staining for discrimination of vascular and tissue leukocytes. Nat. Protoc. *9*, 209–222.

Annunziato, F., Santarlasci, V., Maggi, L., Cosmi, L., Liotta, F., and Romagnani, S. (2013). Reasons for rarity of Th17 cells in inflammatory sites of human disorders. Semin. Immunol. *25*, 299–304.

Atarashi, K., Nishimura, J., Shima, T., Umesaki, Y., Yamamoto, M., Onoue, M., Yagita, H., Ishii, N., Evans, R., Honda, K., and Takeda, K. (2008). ATP drives lamina propria T(H)17 cell differentiation. Nature *455*, 808–812.

Atarashi, K., Tanoue, T., Ando, M., Kamada, N., Nagano, Y., Narushima, S., Suda, W., Imaoka, A., Setoyama, H., Nagamori, T., et al. (2015). Th17 Cell Induction by Adhesion of Microbes to Intestinal Epithelial Cells. Cell *163*, 367–380.

Baeyens, A., Fang, V., Chen, C., and Schwab, S.R. (2015). Exit Strategies: S1P Signaling and T Cell Migration. Trends Immunol. *36*, 778–787.

Benakis, C., Brea, D., Caballero, S., Faraco, G., Moore, J., Murphy, M., Sita, G., Racchumi, G., Ling, L., Pamer, E.G., et al. (2016). Commensal microbiota affects ischemic stroke outcome by regulating intestinal γδ T cells. Nat. Med. *22*, 516–523.

Bollée, G., Flamant, M., Schordan, S., Fligny, C., Rumpel, E., Milon, M., Schordan, E., Sabaa, N., Vandermeersch, S., Galaup, A., et al. (2011). Epidermal growth factor receptor promotes glomerular injury and renal failure in rapidly progressive crescentic glomerulonephritis. Nat. Med. *17*, 1242–1250.

Codarri, L., Gyülvészi, G., Tosevski, V., Hesske, L., Fontana, A., Magnenat, L., Suter, T., and Becher, B. (2011). RORγt drives production of the cytokine GM-CSF in helper T cells, which is essential for the effector phase of autoimmune neuroinflammation. Nat. Immunol. *12*, 560–567.

Collins, J.W., Keeney, K.M., Crepin, V.F., Rathinam, V.A., Fitzgerald, K.A., Finlay, B.B., and Frankel, G. (2014). Citrobacter rodentium: infection, inflammation and the microbiota. Nat. Rev. Microbiol. *12*, 612–623.

(K) Quantification of glomerular crescent formation and tubulointerstitial damage of nephritic *Il17a*$^{-/-}$ mice ± vancomycin-treatment (scale bar, 25 μm).
(L) BUN and albuminuria in *Il17a*$^{-/-}$ ± vancomycin-treatment.
(M) Quantification of renal neutrophil recruitment 10 days after nephritis induction.
Data are representative of three independent experiments. Symbols represent individual data points, with the mean as a horizontal line. *$p < 0.05$, **$p < 0.01$, ***$p < 0.001$, ****$p < 0.0001$.
See also Figure S7.

Couser, W.G. (2012). Basic and translational concepts of immune-mediated glomerular diseases. J. Am. Soc. Nephrol. *23*, 381–399.

Disteldorf, E.M., Krebs, C.F., Paust, H.J., Turner, J.E., Nouailles, G., Tittel, A., Meyer-Schwesinger, C., Stege, G., Brix, S., Velden, J., et al. (2015). CXCL5 drives neutrophil recruitment in TH17-mediated GN. J. Am. Soc. Nephrol. *26*, 55–66.

Esplugues, E., Huber, S., Gagliani, N., Hauser, A.E., Town, T., Wan, Y.Y., O'Connor, W., Jr., Rongvaux, A., Van Rooijen, N., Haberman, A.M., et al. (2011). Control of TH17 cells occurs in the small intestine. Nature *475*, 514–518.

Gaffen, S.L., Jain, R., Garg, A.V., and Cua, D.J. (2014). The IL-23-IL-17 immune axis: from mechanisms to therapeutic testing. Nat. Rev. Immunol. *14*, 585–600.

Gan, P.Y., Steinmetz, O.M., Tan, D.S., O'Sullivan, K.M., Ooi, J.D., Iwakura, Y., Kitching, A.R., and Holdsworth, S.R. (2010). Th17 cells promote autoimmune anti-myeloperoxidase glomerulonephritis. J. Am. Soc. Nephrol. *21*, 925–931.

Harrington, L.E., Hatton, R.D., Mangan, P.R., Turner, H., Murphy, T.L., Murphy, K.M., and Weaver, C.T. (2005). Interleukin 17-producing CD4+ effector T cells develop via a lineage distinct from the T helper type 1 and 2 lineages. Nat. Immunol. *6*, 1123–1132.

Hirota, K., Duarte, J.H., Veldhoen, M., Hornsby, E., Li, Y., Cua, D.J., Ahlfors, H., Wilhelm, C., Tolaini, M., Menzel, U., et al. (2011). Fate mapping of IL-17-producing T cells in inflammatory responses. Nat. Immunol. *12*, 255–263.

Hirota, K., Turner, J.E., Villa, M., Duarte, J.H., Demengeot, J., Steinmetz, O.M., and Stockinger, B. (2013). Plasticity of Th17 cells in Peyer's patches is responsible for the induction of T cell-dependent IgA responses. Nat. Immunol. *14*, 372–379.

Horai, R., Zárate-Bladés, C.R., Dillenburg-Pilla, P., Chen, J., Kielczewski, J.L., Silver, P.B., Jittayasothorn, Y., Chan, C.C., Yamane, H., Honda, K., and Caspi, R.R. (2015). Microbiota-Dependent Activation of an Autoreactive T Cell Receptor Provokes Autoimmunity in an Immunologically Privileged Site. Immunity *43*, 343–353.

Hünemörder, S., Treder, J., Ahrens, S., Schumacher, V., Paust, H.J., Menter, T., Matthys, P., Kamradt, T., Meyer-Schwesinger, C., Panzer, U., et al. (2015). TH1 and TH17 cells promote crescent formation in experimental autoimmune glomerulonephritis. J. Pathol. *237*, 62–71.

Ivanov, I.I., McKenzie, B.S., Zhou, L., Tadokoro, C.E., Lepelley, A., Lafaille, J.J., Cua, D.J., and Littman, D.R. (2006). The orphan nuclear receptor RORgammat directs the differentiation program of proinflammatory IL-17+ T helper cells. Cell *126*, 1121–1133.

Ivanov, I.I., Frutos, Rde.L., Manel, N., Yoshinaga, K., Rifkin, D.B., Sartor, R.B., Finlay, B.B., and Littman, D.R. (2008). Specific microbiota direct the differentiation of IL-17-producing T-helper cells in the mucosa of the small intestine. Cell Host Microbe *4*, 337–349.

Ivanov, I.I., Atarashi, K., Manel, N., Brodie, E.L., Shima, T., Karaoz, U., Wei, D., Goldfarb, K.C., Santee, C.A., Lynch, S.V., et al. (2009). Induction of intestinal Th17 cells by segmented filamentous bacteria. Cell *139*, 485–498.

Kallal, L.E., Schaller, M.A., Lindell, D.M., Lira, S.A., and Lukacs, N.W. (2010). CCL20/CCR6 blockade enhances immunity to RSV by impairing recruitment of DC. Eur. J. Immunol. *40*, 1042–1052.

Kitching, A.R., and Holdsworth, S.R. (2011). The emergence of TH17 cells as effectors of renal injury. J. Am. Soc. Nephrol. *22*, 235–238.

Krebs, C.F., Kapffer, S., Paust, H.J., Schmidt, T., Bennstein, S.B., Peters, A., Stege, G., Brix, S.R., Meyer-Schwesinger, C., Müller, R.U., et al. (2013). MicroRNA-155 drives TH17 immune response and tissue injury in experimental crescentic GN. J. Am. Soc. Nephrol. *24*, 1955–1965.

Kursar, M., Jänner, N., Pfeffer, K., Brinkmann, V., Kaufmann, S.H., and Mittrücker, H.W. (2008). Requirement of secondary lymphoid tissues for the induction of primary and secondary T cell responses against Listeria monocytogenes. Eur. J. Immunol. *38*, 127–138.

Kurts, C., Panzer, U., Anders, H.J., and Rees, A.J. (2013). The immune system and kidney disease: basic concepts and clinical implications. Nat. Rev. Immunol. *13*, 738–753.

Lee, Y.K., Menezes, J.S., Umesaki, Y., and Mazmanian, S.K. (2011). Proinflammatory T-cell responses to gut microbiota promote experimental autoimmune encephalomyelitis. Proc. Natl. Acad. Sci. USA *108* (*Suppl 1*), 4615–4622.

Leonardi, C., Matheson, R., Zachariae, C., Cameron, G., Li, L., Edson-Heredia, E., Braun, D., and Banerjee, S. (2012). Anti-interleukin-17 monoclonal antibody ixekizumab in chronic plaque psoriasis. N. Engl. J. Med. *366*, 1190–1199.

Mackley, E.C., Houston, S., Marriott, C.L., Halford, E.E., Lucas, B., Cerovic, V., Filbey, K.J., Maizels, R.M., Hepworth, M.R., Sonnenberg, G.F., et al. (2015). CCR7-dependent trafficking of RORγ^{+} ILCs creates a unique microenvironment within mucosal draining lymph nodes. Nat. Commun. *6*, 5862.

Mease, P.J., Genovese, M.C., Greenwald, M.W., Ritchlin, C.T., Beaulieu, A.D., Deodhar, A., Newmark, R., Feng, J., Erondu, N., and Nirula, A. (2014). Brodalumab, an anti-IL17RA monoclonal antibody, in psoriatic arthritis. N. Engl. J. Med. *370*, 2295–2306.

Morton, A.M., Sefik, E., Upadhyay, R., Weissleder, R., Benoist, C., and Mathis, D. (2014). Endoscopic photoconversion reveals unexpectedly broad leukocyte trafficking to and from the gut. Proc. Natl. Acad. Sci. USA *111*, 6696–6701.

Mosmann, T.R., Cherwinski, H., Bond, M.W., Giedlin, M.A., and Coffman, R.L. (1986). Two types of murine helper T cell clone. I. Definition according to profiles of lymphokine activities and secreted proteins. J. Immunol. *136*, 2348–2357.

Nagai, T., Abe, A., and Sasakawa, C. (2005). Targeting of enteropathogenic Escherichia coli EspF to host mitochondria is essential for bacterial pathogenesis: critical role of the 16th leucine residue in EspF. J. Biol. Chem. *280*, 2998–3011.

Nurieva, R., Yang, X.O., Martinez, G., Zhang, Y., Panopoulos, A.D., Ma, L., Schluns, K., Tian, Q., Watowich, S.S., Jetten, A.M., and Dong, C. (2007). Essential autocrine regulation by IL-21 in the generation of inflammatory T cells. Nature *448*, 480–483.

O'Shea, J.J., and Paul, W.E. (2010). Mechanisms underlying lineage commitment and plasticity of helper CD4+ T cells. Science *327*, 1098–1102.

Ooi, J.D., Phoon, R.K., Holdsworth, S.R., and Kitching, A.R. (2009). IL-23, not IL-12, directs autoimmunity to the Goodpasture antigen. J. Am. Soc. Nephrol. *20*, 980–989.

Park, H., Li, Z., Yang, X.O., Chang, S.H., Nurieva, R., Wang, Y.H., Wang, Y., Hood, L., Zhu, Z., Tian, Q., and Dong, C. (2005). A distinct lineage of CD4 T cells regulates tissue inflammation by producing interleukin 17. Nat. Immunol. *6*, 1133–1141.

Paust, H.J., Turner, J.E., Steinmetz, O.M., Peters, A., Heymann, F., Hölscher, C., Wolf, G., Kurts, C., Mittrücker, H.W., Stahl, R.A., and Panzer, U. (2009). The IL-23/Th17 axis contributes to renal injury in experimental glomerulonephritis. J. Am. Soc. Nephrol. *20*, 969–979.

Paust, H.J., Turner, J.E., Riedel, J.H., Disteldorf, E., Peters, A., Schmidt, T., Krebs, C., Velden, J., Mittrücker, H.W., Steinmetz, O.M., et al. (2012). Chemokines play a critical role in the cross-regulation of Th1 and Th17 immune responses in murine crescentic glomerulonephritis. Kidney Int. *82*, 72–83.

Paust, H.J., Riedel, J.H., Krebs, C.F., Turner, J.E., Brix, S.R., Krohn, S., Velden, J., Wiech, T., Kaffke, A., Peters, A., et al. (2015). CXCR3+ Regulatory T Cells Control TH1 Responses in Crescentic GN. J Am Soc Nephrol.

Pisitkun, P., Ha, H.L., Wang, H., Claudio, E., Tivy, C.C., Zhou, H., Mayadas, T.N., Illei, G.G., and Siebenlist, U. (2012). Interleukin-17 cytokines are critical in development of fatal lupus glomerulonephritis. Immunity *37*, 1104–1115.

Rakoff-Nahoum, S., Paglino, J., Eslami-Varzaneh, F., Edberg, S., and Medzhitov, R. (2004). Recognition of commensal microflora by toll-like receptors is required for intestinal homeostasis. Cell *118*, 229–241.

Ramani, K., Pawaria, S., Maers, K., Huppler, A.R., Gaffen, S.L., and Biswas, P.S. (2014). An essential role of interleukin-17 receptor signaling in the development of autoimmune glomerulonephritis. J. Leukoc. Biol. *96*, 463–472.

Riedel, J.H., Paust, H.J., Krohn, S., Turner, J.E., Kluger, M.A., Steinmetz, O.M., Krebs, C.F., Stahl, R.A., and Panzer, U. (2016). IL-17F Promotes Tissue Injury in Autoimmune Kidney Diseases. J. Am. Soc. Nephrol. Published online March 30, 2016. http://dx.doi.org/10.1681/ASN.2015101077.

Sano, T., Huang, W., Hall, J.A., Yang, Y., Chen, A., Gavzy, S.J., Lee, J.Y., Ziel, J.W., Miraldi, E.R., Domingos, A.I., et al. (2015). An IL-23R/IL-22 Circuit Regulates Epithelial Serum Amyloid A to Promote Local Effector Th17 Responses. Cell *163*, 381–393.

Stegeman, C.A., Tervaert, J.W., de Jong, P.E., and Kallenberg, C.G.; Dutch Co-Trimoxazole Wegener Study Group (1996). Trimethoprim-sulfamethoxazole (co-trimoxazole) for the prevention of relapses of Wegener's granulomatosis. N. Engl. J. Med. *335*, 16–20.

Steinhoff, U., Brinkmann, V., Klemm, U., Aichele, P., Seiler, P., Brandt, U., Bland, P.W., Prinz, I., Zügel, U., and Kaufmann, S.H. (1999). Autoimmune intestinal pathology induced by hsp60-specific CD8 T cells. Immunity *11*, 349–358.

Steinmetz, O.M., Summers, S.A., Gan, P.Y., Semple, T., Holdsworth, S.R., and Kitching, A.R. (2011). The Th17-defining transcription factor RORγt promotes glomerulonephritis. J. Am. Soc. Nephrol. *22*, 472–483.

Summers, S.A., Steinmetz, O.M., Li, M., Kausman, J.Y., Semple, T., Edgtton, K.L., Borza, D.B., Braley, H., Holdsworth, S.R., and Kitching, A.R. (2009). Th1 and Th17 cells induce proliferative glomerulonephritis. J. Am. Soc. Nephrol. *20*, 2518–2524.

Tomura, M., Honda, T., Tanizaki, H., Otsuka, A., Egawa, G., Tokura, Y., Waldmann, H., Hori, S., Cyster, J.G., Watanabe, T., et al. (2010). Activated regulatory T cells are the major T cell type emigrating from the skin during a cutaneous immune response in mice. J. Clin. Invest. *120*, 883–893.

Tsuboi, N., Asano, K., Lauterbach, M., and Mayadas, T.N. (2008). Human neutrophil Fcgamma receptors initiate and play specialized nonredundant roles in antibody-mediated inflammatory diseases. Immunity *28*, 833–846.

Tulone, C., Giorgini, A., Freeley, S., Coughlan, A., and Robson, M.G. (2011). Transferred antigen-specific T(H)17 but not T(H)1 cells induce crescentic glomerulonephritis in mice. Am. J. Pathol. *179*, 2683–2690.

Turner, J.E., Paust, H.J., Steinmetz, O.M., Peters, A., Riedel, J.H., Erhardt, A., Wegscheid, C., Velden, J., Fehr, S., Mittrücker, H.W., et al. (2010). CCR6 recruits regulatory T cells and Th17 cells to the kidney in glomerulonephritis. J. Am. Soc. Nephrol. *21*, 974–985.

Wu, H.J., Ivanov, I.I., Darce, J., Hattori, K., Shima, T., Umesaki, Y., Littman, D.R., Benoist, C., and Mathis, D. (2010). Gut-residing segmented filamentous bacteria drive autoimmune arthritis via T helper 17 cells. Immunity *32*, 815–827.

Yang, Y., Torchinsky, M.B., Gobert, M., Xiong, H., Xu, M., Linehan, J.L., Alonzo, F., Ng, C., Chen, A., Lin, X., et al. (2014). Focused specificity of intestinal TH17 cells towards commensal bacterial antigens. Nature *510*, 152–156.

Zenewicz, L.A., Yancopoulos, G.D., Valenzuela, D.M., Murphy, A.J., Karow, M., and Flavell, R.A. (2007). Interleukin-22 but not interleukin-17 provides protection to hepatocytes during acute liver inflammation. Immunity *27*, 647–659.

Immunity, Volume *45*

Supplemental Information

Autoimmune Renal Disease Is Exacerbated

by S1P-Receptor-1-Dependent Intestinal

Th17 Cell Migration to the Kidney

Christian F. Krebs, Hans-Joachim Paust, Sonja Krohn, Tobias Koyro, Silke R. Brix, Jan-Hendrik Riedel, Patricia Bartsch, Thorsten Wiech, Catherine Meyer-Schwesinger, Jiabin Huang, Nicole Fischer, Philipp Busch, Hans-Willi Mittrücker, Ulrich Steinhoff, Brigitta Stockinger, Laura Garcia Perez, Ulrich O. Wenzel, Matthias Janneck, Oliver M. Steinmetz, Nicola Gagliani, Rolf A.K. Stahl, Samuel Huber, Jan-Eric Turner, and Ulf Panzer

Patients	Age (years)	Sex	ANCA type	Creatinine (mg/dL)	Proteinuria	Immunosuppression prior to biopsy
1	73	f	pANCA (MPO)	0.7	0.8 g/d	Steroids
2	41	m	cANCA (PR3)	1.2	1.0 g/d	MTX / Steroids
3	51	m	pANCA (MPO)	1.6	0.8 ratio	None
4	63	m	pANCA (MPO)	1.8	1 ratio	AZA / Steroids
5	74	m	pANCA (MPO)	2.6	0.5 ratio	None
6	66	m	pANCA (MPO)	9.1	1.5 ratio	Steroids
7	55	f	pANCA (MPO)	1.6	NA	RTX / Steroids
8	64	m	cANCA (PR3)	3.2	0.25 g/d	AZA / Steroids

Figure S1. Related to Figure 1. Baseline characteristics of patients with ANCA-GN at the time of biopsy. ANCA-GN = anti-neutrophil cytoplasmic antibody-associated glomerulonephritis; pANCA = perinuclear ANCA; MPO = myeloperoxidase; cANCA = cytoplasmic ANCA; PR3 = proteinase 3; m = male; f = female.; MTX = methotrexate; AZA = azathioprin; RTX = Rituximab; NA = not available.

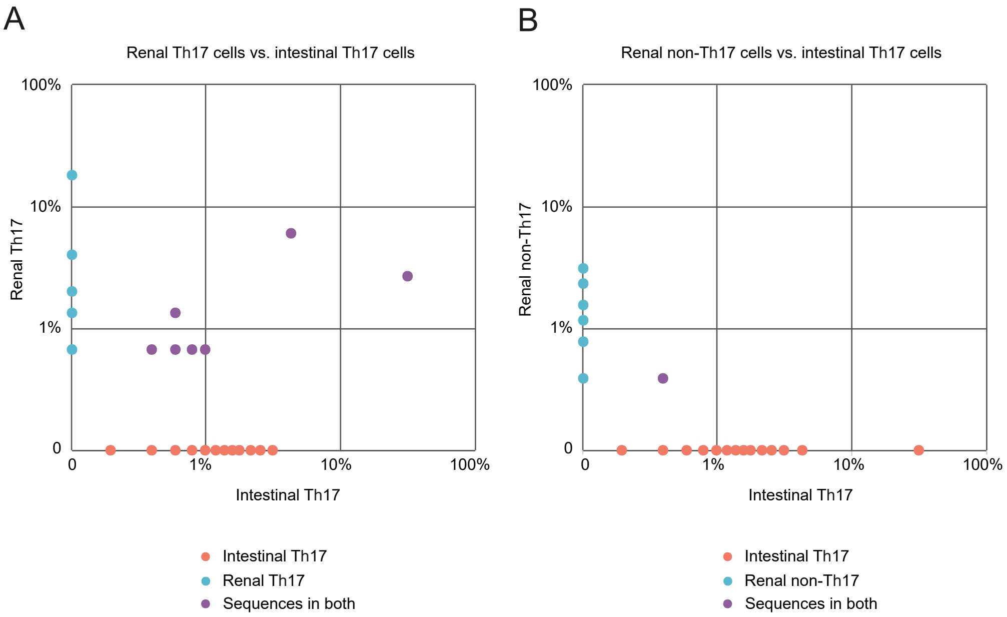

Figure S2. Related to Figure 2. Renal Th17 cells share more *TCRβ* sequences with intestinal Th17 cells than renal non-Th17 cells. cGN was induced in IL-17A-fate reporter mice (*Il17a*^{Cre} x *R26R*^{eYFP}). At day 10 renal Th17 cells (YFP^{+}), renal non-Th17 cells (YFP^{-}) and small intestinal Th17 cells (YFP^{+}) were FACS sorted, DNA was extracted and samples were analyzed for *TCRβ* sequences using the ImmunoSEQ platform (n=3). (A) Representative dot-plot diagram showing the percentage of individual clones in intestinal Th17 cells (orange), renal Th17 cells (blue) and those present in both populations (violet). (B) Dot plot showing the percentage of individual clones in intestinal Th17 cells (orange), renal non-Th17 cells (blue) and those present in both populations (violet).

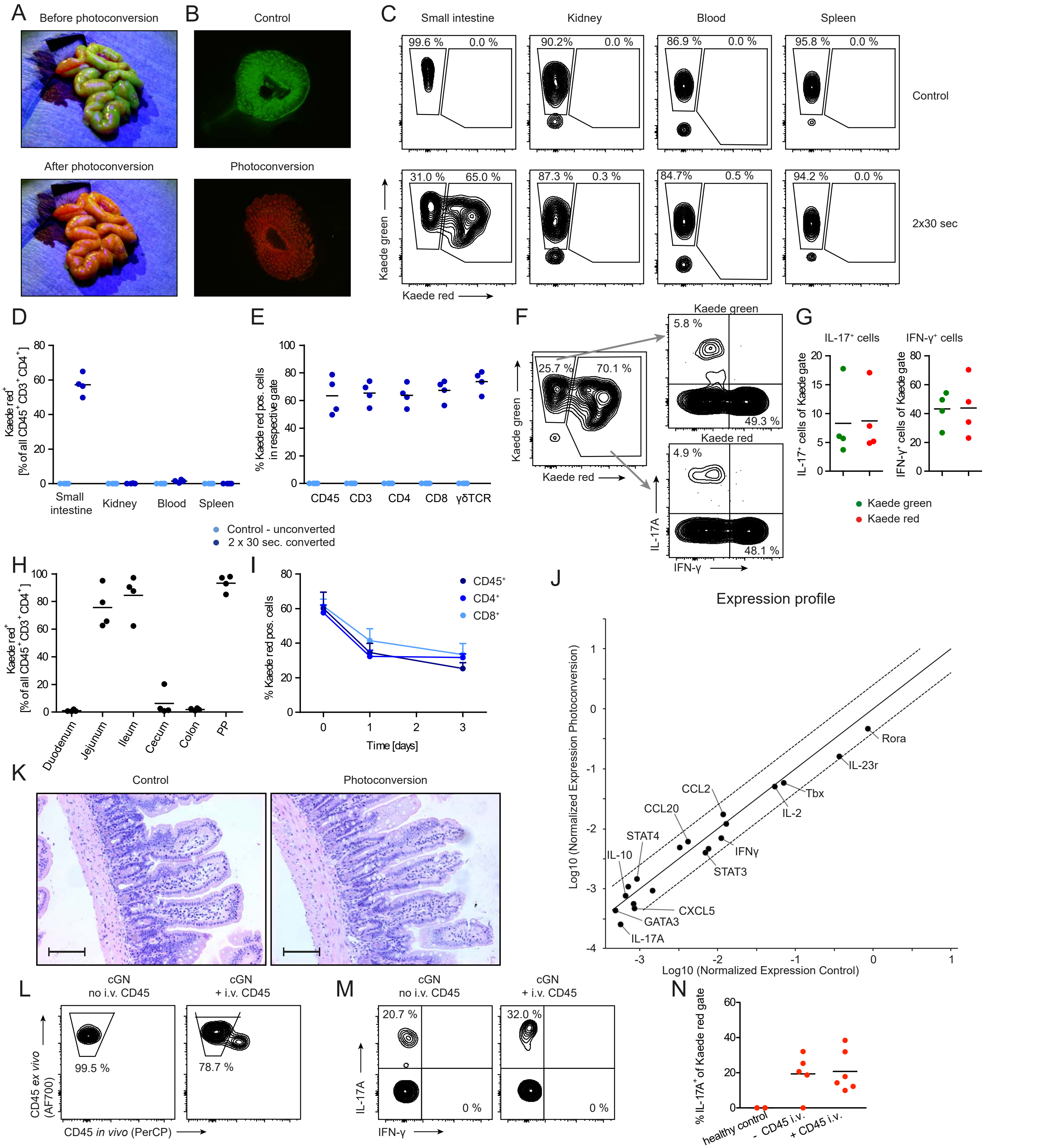

Figure S3. Related to Figure 3. Labeling of intestinal cells in photoconvertible *Kaede*-transgenic mice. (A) Photographs before and after photoconversion of cells in the small intestine using *Kaede* mice. (B) Confocal microscopy of the small intestine before and after photoconversion in *Kaede* mice. (C) Flow cytometry for Kaede green (unconverted) and Kaede red (converted) cells of small intestine lamina propria, kidney, blood and spleen from *Kaede*-tg mice directly after photoconversion of cells in the small intestine and *Kaede*-tg mice without photoconversion (control). (D) Quantification of photoconversion rate in CD3$^+$CD4$^+$ T cells (Kaede red$^+$ cells) in *Kaede*-tg mice directly after photoconversion of cells in the small intestine (dark blue) and *Kaede*-tg control (light blue). (E) Rate of photoconversion (Kaede red$^+$) in CD45, CD3, CD4, CD8 and γδ T cells from small intestine after photoconversion of the small intestine (dark blue) and control (light blue). (F) Kaede green$^+$ and Kaede red$^+$ were analyzed for IL-17A and IFN-γ expression. (G) Quantification of IL-17A and IFN-γ in Kaede green$^+$ and Kaede red$^+$ cells. (H) Quantification of the distribution of Kaede red$^+$ cells in the intestine directly after photoconversion of the small intestine in *Kaede*-tg mice. (I) Pulse-chase analysis of Kaede red$^+$ cells in the small intestine after photoconversion of the small intestine in *Kaede*-tg mice (n=3-4 per group). (J) Cytokine and chemokine expression profile from *Kaede*-tg mice 1 day after photoconverion of the small intestine and untouched control mice. (K) HE staining of jejunum of *Kaede*-tg mice 1 day after photoconversion of the small intestine and of untouched control mice. To discriminate between renal and intravascular T cells, mice recieved 5 µg PerCP labelled CD45 antibodies intraveneously 3 min prior to analysis. (L) Analysis of CD4$^+$ T cells from the kidney. (M+N) Analysis of renal CD4$^+$ T cells for IL-17 and IFN-γ expression in mice +/- intraveneously CD45-PerCP. Data are representative of three independent experiments. Scale bar 200 µm.

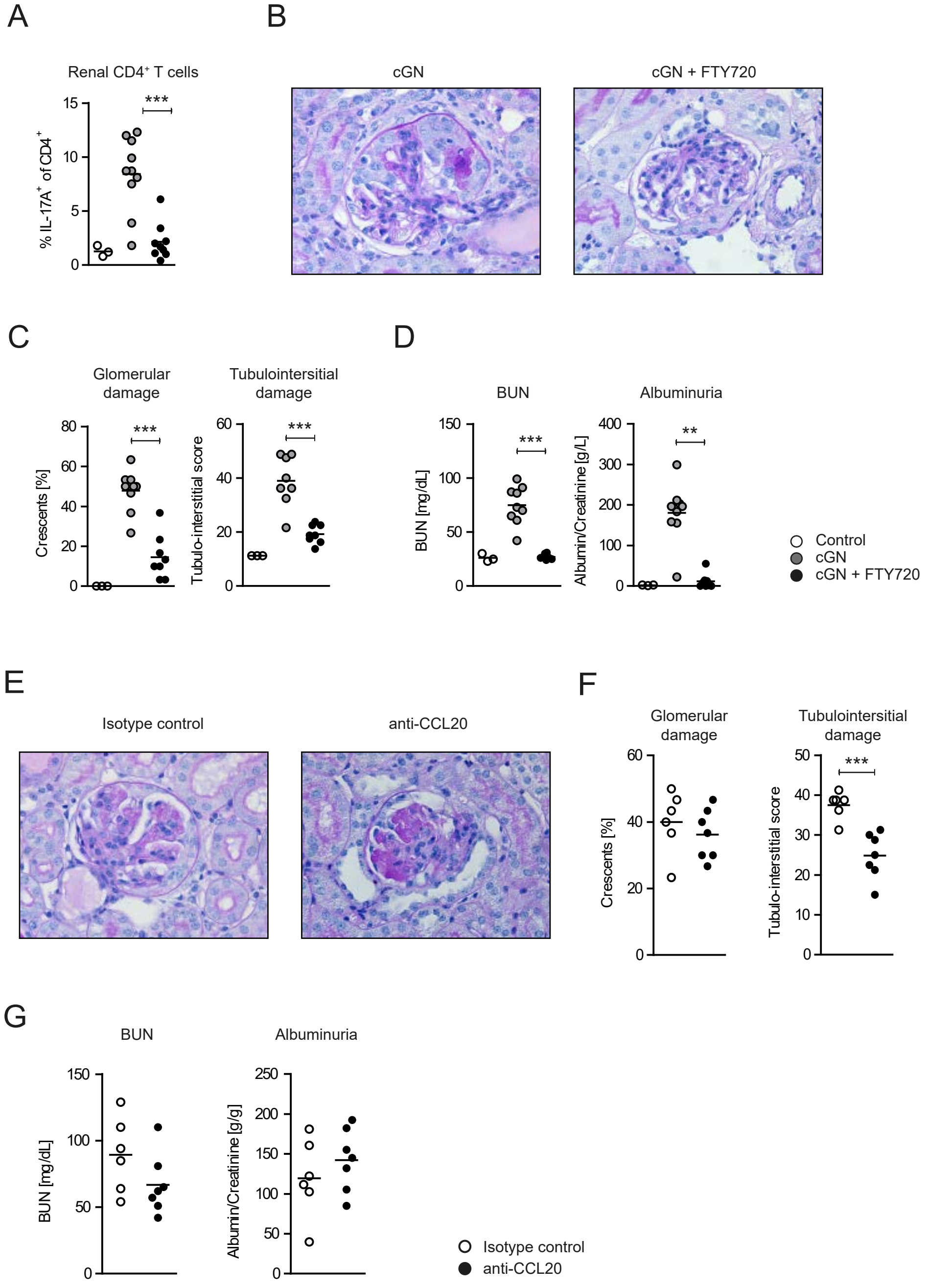

Figure S4. Related to Figure 4. FTY720 reduces renal Th17 cell infiltration and subsequent tissue injury in cGN while CCL20 neutralization did not affect the clinical cours of cGN. (A) After induction of cGN mice were treated with FTY720 via drinking water and analyzed for Th17 cells in the kidney at day 8. (B) PAS-stained tissue sections of renal cortex. (C) Quantification of renal tissue damage (glomerular crescents and tubulointerstitial score) and (D) kidney function (blood urea nitrogen, BUN and albuminuria) were quantified. (E-G) cGN was induced in mice. From day 4 until day 7 mice received anti-CCL20 mab or isotype control. Photoconversion was performed at day 4. (E) PAS-stained tissue sections of renal cortex. (F) Renal tissue damage was quantified (glomerular crescents and tubulointerstitial score) and (G) renal function was measured (blood urea nitrogen, BUN and albuminuria). Data are representative of two (A-D) or three (E-G) independent experiments. ** P<0.01, *** P<0.001.

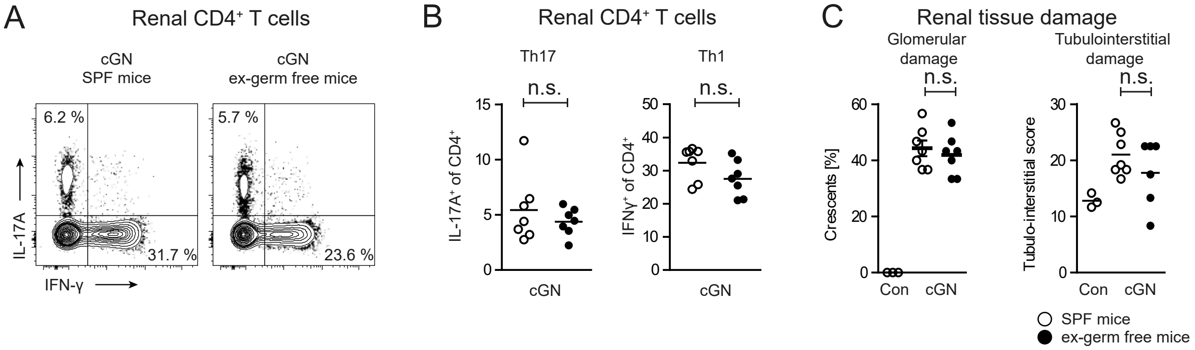

Figure S5. Related to Figure 5. Renal Th17 response was not different between nephritic mice and conventionally colonized ex-germ free mice. (A) IL-17A and IFN-γ expression by flow cytometry of renal $CD4^+$ T cells after induction of cGN in SPF mice and ex-germ free mice reconstituted with SPF microbiota. (B) Quantification of IL-17A and IFN-γ expression. (C) Quantification of glomerular crescent formation and tubulointerstitial damage. Data are representative of two independent experiments. Symbols represent individual data points with the mean as a horizontal line.

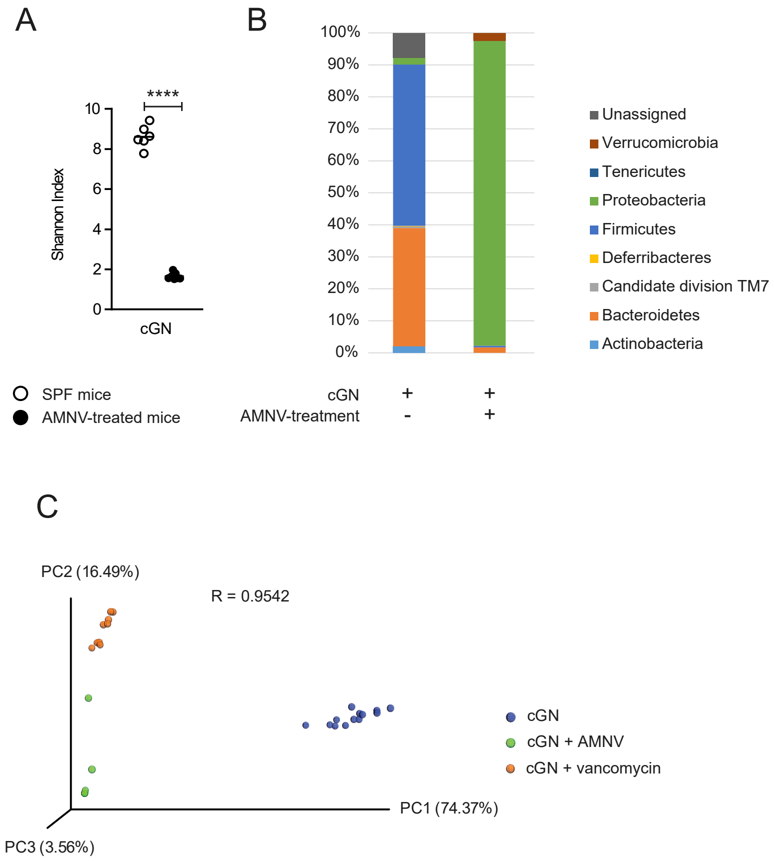

Figure S6. Related to Figure 5. Effect of antibiotic treatment on intestinal microbiota. (A) Analysis of alpha diversity (shannon index) of microbiota from large intestine of mice with AMNV-treatment (ampicillin, metronidazole, neomycin, vancomycin) and SPF control mice. (B) Abundance of bacteria on phylum level in mice after AMNV-treatment and control. (C) Beta diversity analysis of the datasets. 3D Principle component analysis plots showing the relation of the datasets using weighted UniFrac metric. Individual datasets are represented as colored spheres. Analysis of similarity (ANOSIM) tests were applied showing that the microbiota profiles from the datasets are highly significant (cGN: n=13; cGN + AMNV: n=11; cGN + vancomycin: n=10). Data are representative of two independent experiments. **** $P<0.0001$. The R-statistics was calculated as 0.9542.

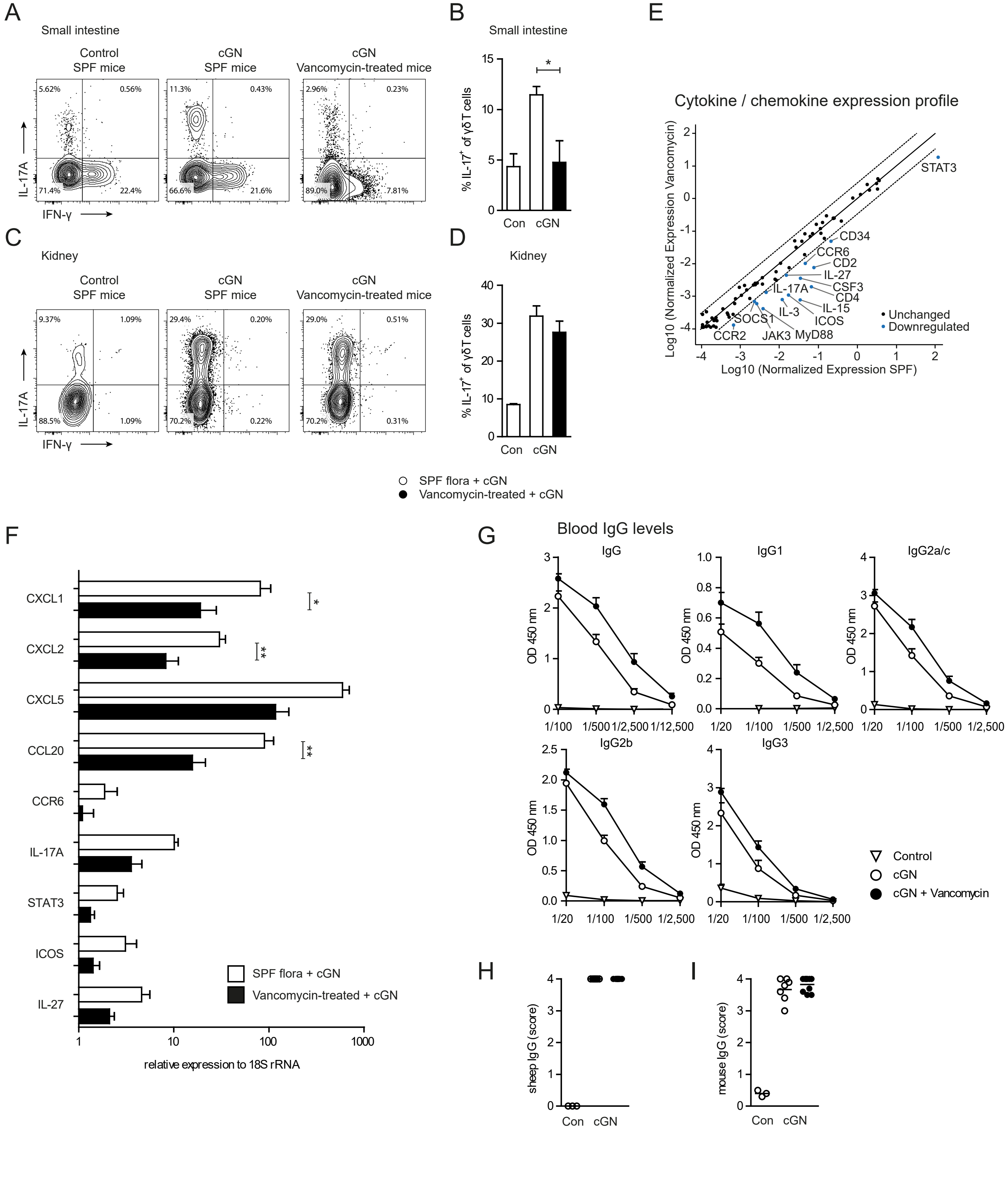

Figure S7. Related to Figure 7. Vancomycin-treatment reduces IL-17A expression by intestinal but not renal γδ T cells and affects renal cytokine and chemokine expression in cGN indendent of the humoral immune response. γδ T cells from the small intestine (A-B) and the kidney (C-D) of mice with cGN were analyzed for IL-17 and IFN-γ expression by flow cytometry. (E+F) Mice were treated for 4 weeks with vancomycin via the drinking water prior to induction of cGN. (E) Cytokine and chemokine expression profile of renal cortex from SPF mice with cGN and vancomycin-treated mice with cGN. (F) RT-PCR analysis of renal cortex as indicated (n=5-6 per group). (G) ELISA of circulating serum mouse anti-sheep total IgG-, IgG1-, IgG2a/2c-, IgG2b-, and IgG3-levels at different dilutions from controls (n=3), nephritic wildtype (n=7), and vancomycin treated nephritic mice (n=10) ten days after induction of cGN. (H) Quantification of glomerular sheep IgG- and (I) glomerular mouse IgG-deposition from controls (n=3), nephritic wildtype (n=7), and vancomycin treated nephritic mice (n=10). Data are representative of three independent experiments. * P<0.05, ** P<0.01.

SUPPLEMENTAL EXPERIMENTAL PROCEDURES

Generation of nephrotoxic serum

To generate nephrotoxic serum, murine renal cortex was homogenized by magnetic beads using a Tissue Lyser II (Qiagen, Hilden, Germany) and sonicated subsequently using a Bandelin Sonopuls (Bandelin, Berlin, Germany) at 100%. Pellets were resuspended in PBS and used for immunization of sheep. Immunization of sheep was performed 4-times within 3 months at Eurogentec (Cologne, Germany). Sheep immunoglobulin fraction was enriched in two precipitation steps with ammonium sulfate at a final concentration of 50% and dialyzed against phosphate buffered saline (PBS).

Morphological analyses

Paraffin-embedded sections (2 µm) were stained with an antibody directed against GR-1 (Ly6 G/C; NIMP-R14, Hycult Biotech, Uden, Netherland). Tubulointerstitial GR-1^+ cells in 20 low-power fields (magnification ×200) were counted. For the evaluation of Kaede photoconversion in cells of the small intestine, tissue sections were subjected to confocal imaging without further preparation. Kidney samples were snap-frozen in Tissue-Tec and stored at -80 °C. Cryosections were cut at 12 mm on a Leica model CM1850 freezing microtome, stained with anti-CD3 and analyzed by confocal imaging. All slides were evaluated under an Axioskop light microscope and photographed with an Axiocam HRc (Zeiss) using the ZEN software or by confocal microscopy with an A1R using NIS-Element software (Nikon).

T cell receptor sequencing

At day 10 after induction of cGN in *Il17a* fate reporter mice ($Il17a^{\text{Cre}}$ x $R26R^{\text{eYFP}}$) renal Th17 cells (YFP^+; 3-7.5 x 10^4 cells), renal non-Th17 cells (YFP^-; 10-50 x 10^4 cells) and small intestinal Th17 cells (YFP^+; 1-2 x 10^4 cells) were FACS sorted. DNA extraction was performed using the QIAamp DNA Micro Kit (Qiagen) according to the manufacturer's instructions. TCRβ-chain sequencing was conducted by Adaptive Biotechnologies (Seattle, WA, USA) based on the ImmunoSEQ platform (http://www.immunoseq.com) (Becattini et al., 2015). Samples with > 80 productive TCR sequences were taken into the final analysis.

16S rRNA amplicon library preparation and MiSeq sequencing

DNA was automatically extracted from mouse feces using QiaSymphony (Qiagen). Briefly, mouse feces were resuspendend in 1ml extraction buffer; 200 µl were used in the extraction protocol and DNA was eluted in 100µl volume. Amplicons were generated using the following degenerate primers containing the Illumina adapter consensus sequence F (5'- TCGTCGGCAGCGTCAGATGTGTATAAGAGACAGCCTACGGG NGGCWGCAG-3') and R (5'-GTCTCGTGGGCTCGGAGATGTGTATAAGAGACAGGACTAC HVGGGTATCTAATCC-3`) as recently described (Klindworth et al., 2013). Detailed description of the protocol is provided by Illumina (http://www.illumina.com/content; 16s-metagenomic-library-prep-guide-15044223-b.pdf). Illumina Nextera XT Index Kit was used for multiplexing. Barcoded libraries were quantified using the Qubit dsDNA HS Assay Kit (Life Technologies) and subsequently pooled. The libraries were sequenced by 2 x 500 bp PE sequencing on the MiSeq platform.

Bioinformatic processing

FastQC (Babraham Bioinformatics, Babraham Institute, UK) was used to analyze the average quality scores of each sample before and after paired reads. The paired ends in each sample were joined and all sequences less than 250 bp and/or with a Phred score <33 were discarded. Further quality filtering was applied using QIIME (Caporaso et al., 2010) (at Phred >= Q20). QIIME version 1.7 was used to perform OTU clustering and alpha and beta diversity analysis. Chimera filter was applied using usearch8.1. All sequences were clustered based on 97% similarity to reference sequences. The reads railing to meet the 97% similarity were clustered de novo at 97% similarity. Taxonomy levels of representative sequences in the OTU table were assigned according to the SILVA database at 95% similarity. Alpha diversity based on Shannon diversity index was calculated. Beta diversity statistics (ANOSIM) was performed to determine whether differences between the distributions of the microbiota profiles from the three datasets were significant.

SUPPLEMENTAL REFERENCES

Becattini, S., Latorre, D., Mele, F., Foglierini, M., De Gregorio, C., Cassotta, A., Fernandez, B., Kelderman, S., Schumacher, T.N., Corti, D., *et al.* (2015). T cell immunity. Functional heterogeneity of human memory CD4(+) T cell clones primed by pathogens or vaccines. Science *347*, 400-406.

Caporaso, J.G., Kuczynski, J., Stombaugh, J., Bittinger, K., Bushman, F.D., Costello, E.K., Fierer, N., Pena, A.G., Goodrich, J.K., Gordon, J.I., *et al.* (2010). QIIME allows analysis of high-throughput community sequencing data. Nat Methods *7*, 335-336.

Klindworth, A., Pruesse, E., Schweer, T., Peplies, J., Quast, C., Horn, M., and Glockner, F.O. (2013). Evaluation of general 16S ribosomal RNA gene PCR primers for classical and next-generation sequencing-based diversity studies. Nucleic Acids Res *41*, e1.
